# Benchmarking transposable element annotation methods for creation of a streamlined, comprehensive pipeline

**DOI:** 10.1186/s13059-019-1905-y

**Published:** 2019-12-16

**Authors:** Shujun Ou, Weija Su, Yi Liao, Kapeel Chougule, Jireh R. A. Agda, Adam J. Hellinga, Carlos Santiago Blanco Lugo, Tyler A. Elliott, Doreen Ware, Thomas Peterson, Ning Jiang, Candice N. Hirsch, Matthew B. Hufford

**Affiliations:** 1 Department of Ecology, Evolution, and Organismal Biology, Iowa State University, Ames, IA 50011 USA; 2 Department of Genetics, Development, and Cell Biology, Iowa State University, Ames, IA 50011 USA; 3 Department of Ecology and Evolutionary Biology, University of California, Irvine, CA 92697 USA; 4 Cold Spring Harbor Laboratory, Cold Spring Harbor, NY 11724 USA; 5 Centre for Biodiversity Genomics, University of Guelph, Guelph, Ontario N1G 2W1 Canada; 6 USDA-ARS NEA Robert W. Holley Center for Agriculture and Health, Cornell University, Ithaca, NY 14853 USA; 7 Department of Horticulture, Michigan State University, East Lansing, MI 48824 USA; 8 Department of Agronomy and Plant Genetics, University of Minnesota, Saint Paul, MN 55108 USA

**Keywords:** Transposable element, Annotation, Genome, Benchmarking, Pipeline

## Abstract

**Background:**

Sequencing technology and assembly algorithms have matured to the point that high-quality de novo assembly is possible for large, repetitive genomes. Current assemblies traverse transposable elements (TEs) and provide an opportunity for comprehensive annotation of TEs. Numerous methods exist for annotation of each class of TEs, but their relative performances have not been systematically compared. Moreover, a comprehensive pipeline is needed to produce a non-redundant library of TEs for species lacking this resource to generate whole-genome TE annotations.

**Results:**

We benchmark existing programs based on a carefully curated library of rice TEs. We evaluate the performance of methods annotating long terminal repeat (LTR) retrotransposons, terminal inverted repeat (TIR) transposons, short TIR transposons known as miniature inverted transposable elements (MITEs), and Helitrons. Performance metrics include sensitivity, specificity, accuracy, precision, FDR, and *F*_1_. Using the most robust programs, we create a comprehensive pipeline called Extensive *de-novo* TE Annotator (EDTA) that produces a filtered non-redundant TE library for annotation of structurally intact and fragmented elements. EDTA also deconvolutes nested TE insertions frequently found in highly repetitive genomic regions. Using other model species with curated TE libraries (maize and Drosophila), EDTA is shown to be robust across both plant and animal species.

**Conclusions:**

The benchmarking results and pipeline developed here will greatly facilitate TE annotation in eukaryotic genomes. These annotations will promote a much more in-depth understanding of the diversity and evolution of TEs at both intra- and inter-species levels. EDTA is open-source and freely available: https://github.com/oushujun/EDTA.

## Background

Transposable elements (TEs) are repetitive, mobile sequences found in most eukaryotic genomes analyzed to date. Originally discovered by Barbara McClintock in maize (*Zea mays*) [1], TEs are now known to comprise the majority of genetic material in many eukaryotic genomes. For example, TEs make up nearly half of the human (*Homo sapiens*) genome [2] and approximately 85% of the genomes of wheat (*Triticum aestivum*) and maize [3, 4]. The functional and evolutionary significance of TEs has also become increasingly clear. *Stowaway* and *PIF*/*Harbinger* transposons in rice (*Oryza sativa*), for instance, are associated with subspecies-specific hotspots of recombination [5], and specific TE insertions have been associated with plant architecture [6] and flowering time [7] in maize, generating phenotypic variation important during domestication and temperate adaptation.

Despite their prevalence and significance, TEs have remained poorly annotated and studied in all but a few model systems. Transposable elements create a particularly challenging genome assembly problem due to both their high copy number and the complex nesting structures produced by new TE insertions into existing TE sequences. While the low-copy, genic fraction of genomes has assembled well, even with short-read sequencing technology, assemblies of TEs and other repeats have remained incomplete and highly fragmented until quite recently.

Long-read sequencing (e.g., PacBio and Oxford Nanopore) and assembly scaffolding (e.g., Hi-C and BioNano) techniques have progressed rapidly within the last few years. These innovations have been critical for high-quality assembly of the repetitive fraction of genomes. In fact, Ou et al. [8] demonstrated that the assembly contiguity of repetitive sequences in recent long-read assemblies is even better than traditional BAC-based reference genomes. With these developments, inexpensive and high-quality assembly of an entire genome is now possible. Knowing where features (i.e., genes and TEs) exist in a genome assembly is important information for using these assemblies for biological findings. However, unlike the relatively straightforward and comprehensive pipelines established for gene annotation [9–11], current methods for TE annotation can be piecemeal, can be inaccurate, and are highly specific to classes of transposable elements.

Transposable elements fall into two major classes. Class I elements, also known as retrotransposons, use RNA intermediates in their “copy and paste” mechanism of transposition [12]. Class I elements can be further divided into long terminal repeat (LTR) retrotransposons, as well as those that lack LTRs (non-LTRs), which include long interspersed nuclear elements (LINEs) and short interspersed nuclear elements (SINEs). Structural features of these elements can facilitate automated de novo annotation in a genome assembly. For example, LTR elements have a 5-bp target site duplication (TSD), while non-LTRs have either variable length TSDs or lack TSDs entirely, being instead associated with deletion of flanking sequences upon insertion [13]. There are also standard terminal sequences associated with LTR elements (i.e., 5′-TG…C/G/TA-3′ for LTR-*Copia* and 5′-TG…CA-3′ for LTR-*Gypsy* elements), and non-LTRs often have a terminal poly-A tail at the 3′ end of the element (see [14] for a complete description of structural features of each superfamily).

The second major class of TEs, Class II elements, also known as DNA transposons, use DNA intermediates in their “cut and paste” mechanism of transposition [15]. As with Class I elements, DNA transposons have superfamily-specific structural features that can be used to facilitate an automated identification process [16]. For example, *hAT* elements typically have an 8-bp TSD, 12–28-bp terminal inverted repeat sequence (TIRs) and contain 5′-C/TA…TA/G-3′ terminal sequences. Each Class II superfamily has different structural features that need to be considered when TE annotation programs are being developed and deployed [16, 17]. *Helitrons* are a unique subclass of Class II elements that replicate through a rolling-circle mechanism and, as such, do not generate a TSD sequence and do not have TIRs, but do have a signature 5′-TC…CTRR-3′ terminal sequence and frequently a short GC-rich stem-loop structure near the 3′ end of the element [16, 18, 19].

High-quality TE annotations have been generated for several model species through extensive community efforts and manual curation (e.g., human [2], *Drosophila melanogaster* [20], *Arabidopsis thaliana* [21], rice [22, 23], and maize [4]). However, with numerous reference genome assemblies being generated both within and across species, large-scale manual curation is no longer feasible, and automated annotation of TEs is required. Dozens of programs have been developed for this purpose, and these generally fall into one of three categories [24, 25]. First, general repeat finders identify high copy number sequences in a genome [26–28]. These programs can have high sensitivity for identifying repetitive sequences, but have limited ability to classify them into specific TE superfamilies and can misidentify non-TE features (e.g., high copy number genes). Second, the sequence homology approach [29–32] is quick and takes advantage of prior knowledge (i.e., databases), but is limited by the depth and accuracy of this knowledge and variability across TE sequences. The final approach takes advantage of the structural makeup of classes and superfamilies of TEs for de novo structural annotation [24, 25]. This approach is advantageous in that it is codable and does not rely on repeat databases, therefore being ideal for newly assembled species. However, the approach is limited by the knowledge of the sequence structure of TEs and is often characterized by a high false discovery rate.

While numerous and, in some cases, redundant TE identification methods exist, their performance has not been comprehensively benchmarked, despite recognition that this would be an important exercise [33]. Here, we have gathered a broad set of existing TE annotation software and, using several metrics, have compared each program’s performance to a highly curated TE reference library in rice [34]. Based on our benchmarking results, we propose a comprehensive pipeline for the generation of de novo TE libraries that can then be used for genome annotation. Existing curated TE libraries can also be integrated into this pipeline to create an expanded library with new TE exemplars.

## Results

**Fig. 1:**
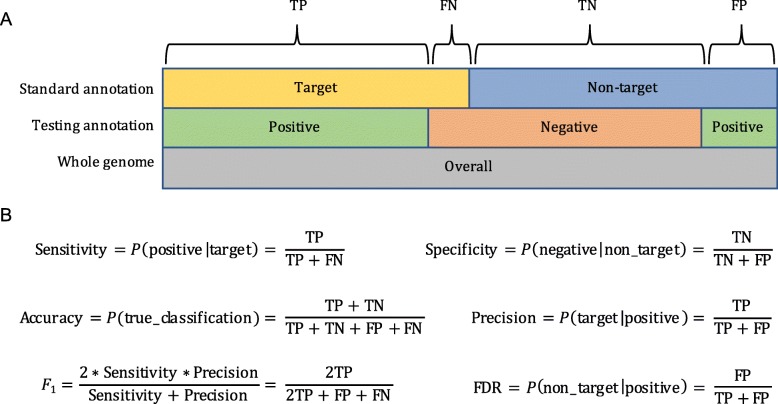
Schematic representation of benchmarking metrics. **a** Definition of TP, true positive; FP, false positive; FN, false negative; and TN, true negative. **b** Definition of sensitivity, specificity, accuracy, precision, *F*_1_ measure, and false discovery rate (FDR). Each metric is calculated based on genomic sequence length in bp

In eukaryotic genomes, transposable elements (TEs) are present as both structurally intact and fragmented sequences. Development of a species-specific TE library is an essential step in the annotation process, which begins with structural identification of major TE classes and can be followed by manual curation. Representative sequences in the library are then used to detect fragmented and mutated TE sequences that are not recognizable using structural features. Importantly, if there are errors in the annotation library, these will be propagated during the whole-genome annotation process. We have benchmarked commonly used programs for metrics including sensitivity, specificity, accuracy, and precision (Fig. 1). To evaluate each program, we used a high-quality, manually curated library developed for the model species *Oryza sativa* (rice), which has a long history of TE discovery and annotation [23, 35–43]. The optimal set of programs determined by this benchmarking have been combined into a comprehensive pipeline called the Extensive *de-novo* TE Annotator (EDTA) [34]. Additionally, the robustness of this pipeline was validated across maize and Drosophila for which high-quality, manually curated TE libraries were available [34].

### Setting up a reference annotation for benchmarking

The reference annotation library for rice was created through substantial manual curation of repeat families obtained from an all-versus-all BLAST search of the rice genome (details in the “Methods” section). This curated library was then used to annotate the rice genome for both structurally intact and fragmented TE sequences, which comprised 23.98% and 22.66% of the rice genome, respectively (46.64% in total; Table 1). Since half of all TEs in the rice genome are fragmented, structural annotation alone would miss a substantial portion of TE sequences. Thus, a homology-based approach that uses a TE library is necessary to obtain a complete annotation. In this study, the whole-genome TE annotation based on the curated library was used as the ground-truth annotation for benchmarking of TE annotation programs.

**Table 1:** TE content in the rice (*Oryza sativa ssp. japonica* cv. “Nipponbare” v. MSU7) genome

	Class	Std6.9.5*	Complete** (%)	Fragmented** (%)	Total** (%)
LTR	Class I	88.1 Mb	14.44	9.11	23.54
Non-LTR	Class I	7.6 Mb	0.51	1.52	2.03
TIR	Class II	65.5 Mb	7.93	9.56	17.49
*Helitron*	Class II	13.4 Mb	1.10	2.47	3.57
Total	–	174.6 Mb	23.98	22.66	46.64

*Annotation based on the curated library (v6.9.5)

**Percent of genome estimated based on a genome size of 374.3 Mb

TEs in this curated library are broken down into a number of non-overlapping categories, including LTR (referring to LTR retrotransposons), non-LTR (including SINEs and LINEs), TIR (referring to DNA transposons with TIRs, including MITEs), *Helitron*, and non-TE repeat sequence. LTR retrotransposons contribute the largest component, 23.54% of the total genomic DNA (Table 1). Non-LTR retrotransposons including SINEs and LINEs contribute the smallest proportion of total sequence (7.6 Mb or ~ 2% of the genome; Table 1). DNA transposons contribute ~ 21% (17.49% TIR elements and 3.57% *Helitrons*; Table 1).

To test various programs, the genome was partitioned into target and non-target sequences (Fig. 1a). For example, when testing the performance of an LTR annotation program, predicted LTR sequences matching our curated library were labeled “target” and all other sequences were labeled “non-target.” Each program’s annotation was then compared to that from our curated library, with sequences included in our target subset counted as true positives (TP), sequences in our non-target subset categorized as false positives (FP), missed targets counted as false negatives (FN), and the remainder of the genome (not TP, FP, nor FN) labeled as true negative (TN; Fig. 1a).

We then used six metrics (sensitivity, specificity, accuracy, precision, FDR, and *F*_1_) to characterize the annotation performance of the test library created by various programs (Fig. 1b). These metrics were calculated based on the total number of genomic DNA bases, because misannotations occurring in the test library will be amplified in the whole-genome annotation process. *Sensitivity* denotes how well the test library can correctly annotate target TE sequences. *Specificity* describes how well the test library can correctly exclude non-target sequences. *Accuracy* denotes the true rate in discriminating target and non-target sequences. *Precision* is the true discovery rate, while *FDR* is the false discovery rate. Finally, the *F*_1_ measure is the harmonic mean of precision and sensitivity; *F*_1_ is similar to accuracy, but is useful because it does not require an estimate of TN, which can be difficult to quantify. While we can estimate TNs with the use of the curated annotation, we still include the *F*_1_ measure in our study to allow for comparison to previous work.

We exhaustively searched the literature for open-source programs and databases that have been developed for general repeat annotations as well as structural annotation programs for LTR elements, SINEs, LINEs, TIR elements, and *Helitrons*. We applied educated parameters based on knowledge of transposon structures to run these programs (see the “Methods” section and Additional file 1). We also applied filters on initial program predictions to remove low-quality candidates and potentially false predictions such as short sequences and tandem-repeat-containing sequences (Additional file 1). For each program, a non-redundant test library was created from filtered TE candidates, which was then used to annotate the rice genome. The annotation from each program for each category of TEs was compared with those from the curated library for calculation of benchmarking metrics.

### Comparison of general repeat annotators

**Fig. 2:**
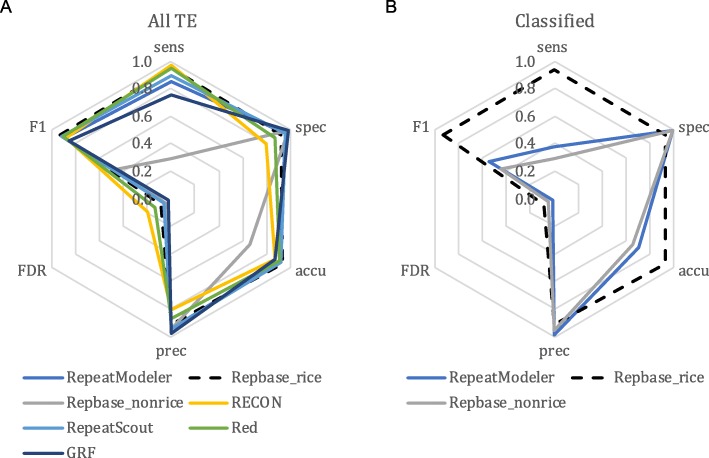
Annotation performance of general repeat annotators compared to the rice curated annotation. **a** Annotation and **b** classification performance of various methods. Sens, sensitivity; Spec, specificity; Accu, accuracy; Prec, precision; FDR, false discovery rate; F1, *F*_1_ measure

**Fig. 3:**
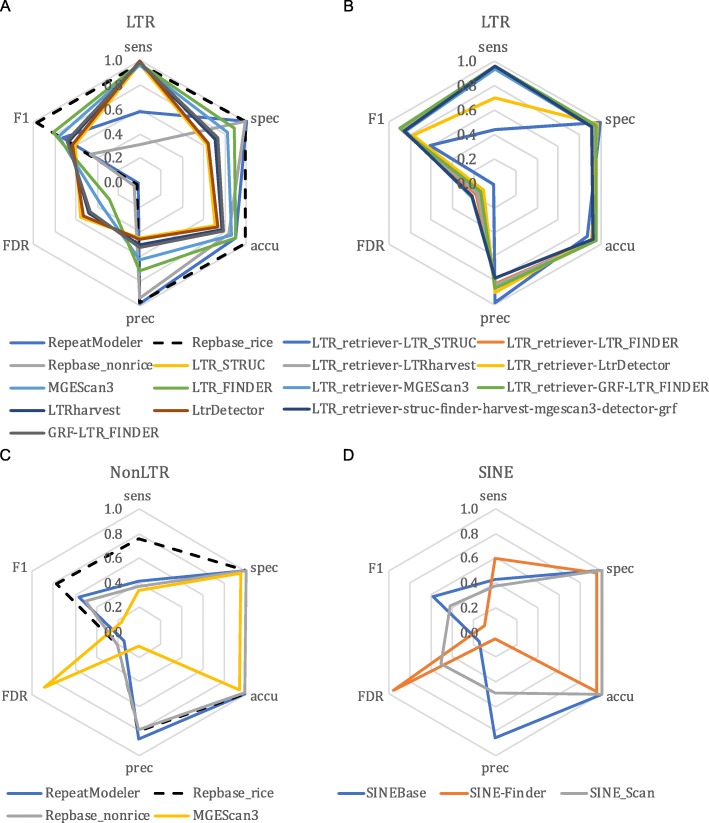
Annotation performance of retrotransposon-related programs as compared to the rice curated annotation. **a** Various methods to identify LTR retrotransposons. GRF-LTR_FINDER combines the terminal direct repeat search engine in GRF and the filtering engine in a modified version of LTR_FINDER for detection of LTR retrotransposons. The LTR_FINDER result was generated by the parallel version. **b** LTR_retriever-specific results, which were generated using LTR_retriever to process results from other programs specified in each of the names in the figure. **c** Non-LTR retrotransposon annotation methods. **d** Short interspersed nuclear element (SINE) annotation methods. Sens, sensitivity; Spec, specificity; Accu, accuracy; Prec, precision; FDR, false discovery rate; F1, *F*_1_ measure

**Fig. 4:**
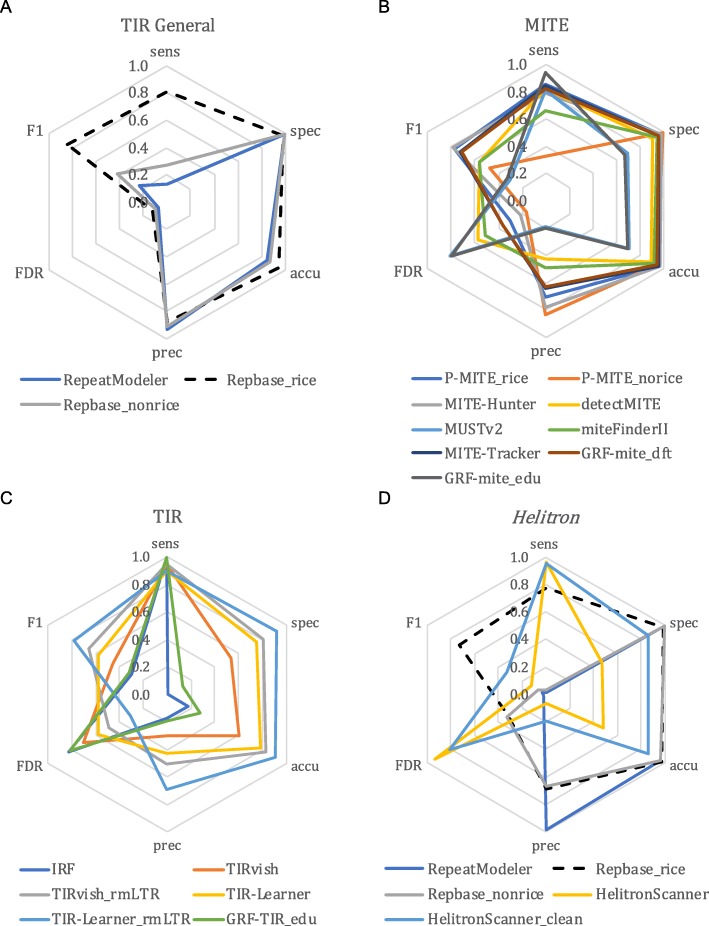
Annotation performance of DNA transposon-related programs as compared to the rice curated annotation. **a** General methods and **c** structure-based methods to identify TIR elements. The TIR-Learner_rmLTR and TIRvish_rmLTR libraries had LTR-related sequences removed using the curated library. **b** Structure-based methods and specialized database to identify miniature inverted transposable elements (MITEs). **d** Annotation performance of *Helitron*-related methods as compared to the rice curated annotation. The HelitronScanner_clean result had non-*Helitron* TE sequences removed using the curated library. Sens, sensitivity; Spec, specificity; Accu, accuracy; Prec, precision; FDR, false discovery rate; F1, *F*_1_ measure

We benchmarked five general repeat annotators, including RECON [44], RepeatScout [26], RepeatModeler [28], Red [27], and Generic Repeat Finder (GRF) [45], as well as a repeat database Repbase [30], which is widely used as the default library in RepeatMasker [29]. For these TE annotation approaches, only RepeatModeler and Repbase provide classification of TE annotations. Among these methods, we found that Repbase employing the rice TE database had very high performance in both TE identification and classification (Fig. 2), which is a product of continuous improvement and curation of rice TEs by the community. However, if we exclude rice-related TEs in Repbase and treat rice as a newly sequenced species (Repbase_norice in Fig. 2), the annotation (Fig. 2a) and classification (Fig. 2b) sensitivity both drop from ~ 94 to ~ 29%, despite extremely high specificity (~ 99%) and low FDR (~ 5%; Additional file 2: Table S1A). This result was consistent for each of the TE classes (Fig. 3a—LTR elements; Fig. 3c—non-LTR elements; Fig. 4a—TIR elements; Fig. 4d—*Helitron*), though the drop in sensitivity was substantially greater for *Helitrons* (dropped from 78 to 3%) than for other elements. For TE classifications, RepeatModeler performed similarly to Repbase without rice sequences (Fig. 2b), and both can, therefore, be used as high-quality supplements to other specialized TE annotators. GRF is the most recently developed general repeat finder. It had the lowest sensitivity (75%; Fig. 2a; Additional file 2: Table S1A), which is likely due to its inability to introduce gaps during the multiple sequence alignment process [45].

Overall, the general repeat finders we tested have consistently high performance in identifying repetitive sequences in the rice genome, with the exception of Repbase without rice sequences (Fig. 2a). What really differentiates these programs is their ease in processing raw results. All are open source and easy to install except Repbase (Additional file 2: Table S2), which requires an institutional subscription for access. Red runs on a single CPU and took the shortest time for execution (~ 33 min); however, Red produced the largest raw result file, which is highly redundant (35 Mb after clustering; Additional file 2: Table S2). RepeatModeler and RepeatScout produced very compact outputs (< 4 Mb). The RepeatScout program runs more efficiently but provides no classification of repeat sequences (Additional file 2: Table S2). The RECON and RepeatScout packages are not actively maintained, but have been incorporated into the RepeatModeler package. In summary, RepeatModeler has the highest performance among the general repeat annotators based on our evaluation metrics (Fig. 2) and is open source, able to produce a compact output, and able to classify TE families to some degree. Still, further classification or use of more specialized software based on the specific structures of each superfamily of TEs is necessary to achieve more accurate annotations.

### Comparison of LTR annotators

LTR retrotransposons have received the most attention in TE annotation software development due to their abundance in eukaryotic genomes. In addition to the two general repeat identification methods with classification (RepeatModeler and Repbase), we found seven structure-based methods that are specifically designed for de novo LTR identification. Chronologically in order of development, they are LTR_STRUC [46], LTR_FINDER [47], LTRharvest [48], MGEScan3 [49], LTR_retriever [40], LtrDetector [50], and GRF [45]. In a previous study [40], we developed LTR_retriever and compared its performance to LTR_STRUC, LTR_FINDER, LTRharvest, and MGEScan_LTR [51]. Here, we update the comparison with the recently developed MGEScan3, LtrDetector, and GRF. Meanwhile, the LTR_retriever package has been updated from v1.6 to v2.7 since its initial publication.

The six structure-based methods that we tested all had very high sensitivity (> 96%) but also high FDR (28–55%); specificity, accuracy, and *F*_1_ measures were also somewhat suboptimal (Fig. 3a). Among these six methods, LTR_FINDER demonstrated the best balance of performance across metrics followed by MGEScan3 (Fig. 3a). However, it runs slowly partly because it is single-threaded. For faster execution of LTR_FINDER, we developed LTR_FINDER_parallel that splits chromosome sequences into shorter segments and executes LTR_FINDER in parallel [52]. We used LTR_FINDER_parallel for all related analyses in this study.

LTR_retriever does not have its own search engine; rather, it was designed as a stringent filtering method for raw results of other LTR programs. LTR_retriever can process results of all six aforementioned LTR methods or any combination of them. We used LTR_retriever in conjunction with each of the six programs and with all six programs together to benchmark its performance. Our results show that LTR_retriever has consistently high specificity (94.8% ± 3%), accuracy (92.2% ± 3%), precision (84.9% ± 7%), and *F*_1_ measure (82.4% ± 10%) and relatively low FDR (15.1% ± 7%) (Fig. 3b; Additional file 2: Table S1B). The sensitivity of LTR_retriever is also high (≥ 93%), except when used in combination with LTR_STRUC and LtrDetector (Fig. 3b; Additional file 2: Table S1B). This is due to the imprecisely defined sequence boundaries of LTR candidates of these two methods, preventing LTR_retriever from finding microstructures like TSD and terminal motifs [40], yielding a high false negative rate.

Overall, LTR_retriever represents the best compromise between sensitivity and specificity. LTR_retriever also generated the most compact LTR library in comparison to the other programs (Additional file 2: Table S2), allowing efficient and precise whole-genome LTR annotations. It is not necessary to run all six structure-based programs along with LTR_retriever. Instead, the combination of LTR_FINDER and LTRharvest with LTR_retriever achieved the best performance and the shortest processing time as previously demonstrated [40].

### Comparison of non-LTR annotators

Non-LTR retrotransposons include LINEs and SINEs that propagate via reverse transcription of RNA intermediates [16]. Identification of non-LTR retrotransposons is very challenging due to the lack of a terminal repeat structure and also their sequences often degenerate quickly [32]. In addition to the general repeat annotators described above, we also benchmarked a dedicated database for SINEs (SINEBase) and three structure-based methods.

SINEBase [32] is a species-agnostic database that performed poorly in terms of sensitivity, similar to the non-rice Repbase library (Fig. 3d). The specialized structure-based annotation methods, including MGEScan3, SINE-Finder, and SINE_Scan also exhibited suboptimal sensitivity (< 60%) and very high FDRs (51–95%) (Fig. 3; Additional file 2: Table S1C). SINE_Scan is a successor of SINE-Finder, which aims to detect all known types of SINEs with higher accuracy [53]. Based on our results, SINE_Scan did have a much lower FDR compared to SINE-Finder; however, its sensitivity was also much lower (Fig. 3d).

The possibility remains that SINEs are under-annotated in the curated library, which may contribute to the high FDR values that were observed across programs. To test the validity of these SINE candidates, we followed the instructions in the SINE_Scan package and manually inspected terminal alignments of all candidate SINE families (*n* = 35). Out of 35 candidate families, we found six longer than 99 bp that possess clear sequence boundaries with poly-A or poly-T tails. These six families were already present in the curated library, indicating the high FDR is a product of false discovery rather than a limitation of the curated library being used to evaluate these programs.

In summary, we found general methods such as RepeatModeler, the non-rice Repbase, and SINEBase provided high-quality annotations for non-LTR retrotransposons, while structure-based methods such as MGEScan3, SINE-Finder, and SINE_Scan have low sensitivity and high rates of false discovery. Therefore, researchers may want to use RepeatModeler for de novo annotation of non-LTR elements, and supplement these annotations with SINEBase or Repbase.

### Comparison of TIR annotators

TIR transposons are a subclass of TEs that carry inverted repeats at their ends [16]. Miniature inverted transposable elements (MITEs) are a special kind of TIR transposon that lack any coding potential (non-autonomous) and are usually shorter than 600 bp [16]. These elements are highly abundant in eukaryotic genomes, and many annotation programs have been designed for their identification. We tested P-MITE [31], a specialized database of curated plant MITEs; IRF [54], TIRvish [55], TIR-Learner [17], and GRF (*grf-main -c 0*) [45], which structurally identify TIR elements; and finally MITE-Hunter [56], detectMITE [57], MUSTv2 [58], miteFinderII [59], MITE-Tracker [60], and GRF (*grf-mite*), which structurally identify MITEs specifically.

The P-MITE database performed similarly to what we observed for classifications from the general repeat annotators; the rice-specific database (P-MITE_rice) annotated TIR elements accurately and sensitively, while the non-rice database (P-MITE_norice) had very low FDR and low sensitivity (Fig. 4b), suggesting the necessity of using structure-based methods for de novo annotation.

We tested four structure-based methods for TIR annotation: IRF, GRF with educated parameters (GRF-TIR_edu), TIRvish, and TIR-Learner. Each of these methods had high sensitivity (> 90%; Fig. 4c; Additional file 2: Table S1D); however, IRF and GRF-TIR_edu performed poorly for the remaining metrics (Fig. 4c). The poor performance of IRF and GRF-TIR_edu is due to the large number of candidates they identified, with 4.7 Gb and 630 Gb (13×–1684× the size of the 374-Mb rice genome) of raw TIR candidate sequences produced, respectively. The majority of raw candidate sequences were overlapping and nested within each other. The output of both programs was substantially filtered and condensed using EDTA utility scripts (Additional file 1; Additional file 2: Table S2), but still had poor performance based on our analysis metrics (Fig. 4c). TIRvish was among the fastest TIR programs (Additional file 2: Table S2); however, it does not provide further classification of superfamilies. In contrast, TIR-Learner provided superfamily classifications and demonstrated relatively high sensitivity, specificity, and accuracy (Fig. 4c), which is promising for TIR annotation.

For structure-based MITE annotation, GRF with educated parameters (GRF-mite_edu) also produced large output files similar to IRF and GRF-TIR_edu. After filtering for false discovery and redundancy (Additional file 1), the candidate sequence file was reduced from 47 Gb (130× the size of the rice genome) to 10 Mb (Additional file 2: Table S2). Still, given its inferior annotation performance relative to other MITE methods (Fig. 4b), GRF-mite_edu is not ideal for de novo annotation. Interestingly, GRF with default parameters (GRF-mite_dft) had high performance similar to MITE-Hunter and MITE-Tracker (Fig. 4b). The poor performance of GRF-mite_edu is mostly due to changing the internal region length from default 780 bp to 10 Kb (Additional file 1), which captured significantly more non-MITE sequences, suggesting the default parameters of GRF may have been optimized for MITE detection. These three MITE methods all had high specificity (≥ 95%) and accuracy (≥ 94%), reasonable sensitivity (79–84%), but somewhat lower precision (64–79%) (Fig. 4b; Additional file 2: Table S1D), suggesting high potential for these programs. miteFinderII and detectMITE also had high performance but with comparatively lower sensitivity for miteFinderII and lower specificity and accuracy for detectMITE (Fig. 4b; Additional file 2: Table S1D). MUSTv2 performed similar to GRF-mite_edu and worse than other MITE programs (Fig. 4b).

We identified promising methods for TIR transposon and MITE annotation including TIR-Learner, MITE-Hunter, MITE-Tracker, and GRF-mite_dft. These methods all have relatively high specificity but somewhat high FDR (Fig. 4), indicating each program generated annotations that matched our curated library as well as additional potential TEs. Our curated library is likely incomplete, and these new candidates could be real TIR elements or MITEs. We compared these new TE candidates with the curated library and to TIR element-specific conserved domains (Additional file 1). On an element basis, we found over 65% (5688 out of 7435 novel TIR elements and 11,885 out of 18,093 novel MITEs) of the candidates shared similar TIR sequences with our curated library, but included more diverse internal sequences, with a subset of elements showing potential to be autonomous (Additional file 3: Table S3). Such variation is common in non-autonomous TIR transposons, such as *Ds* elements [61]. For MITE candidates with novel TIRs, the majority had more than three copies in the rice genome (Additional file 3: Table S3), suggesting these are likely real TEs that were not included in the curated library. Out of the four MITE programs, MITE-Hunter identified sequences most similar to the curated library (Additional file 3: Table S3).

TIR-Learner demonstrated great promise for structural annotation (Fig. 4), and a large proportion of the novel candidates it identified may be non-autonomous forms of known TIR elements (Additional file 3: Table S3). Among the novel TIR elements with novel TIRs, less than half had more than three copies in the rice genome (Additional file 3: Table S3). This is because TIR-Learner does not impose a copy number filter [17], given that some TEs may share similar TIRs but different internal regions (Additional file 3: Table S3). Still, some of these low-copy candidates could be contaminants such as misclassified LTR sequences. In fact, comparison to the curated library showed that 6.38% of TIR-Learner reported TIR candidates were actually LTR sequences. After removal of these contaminants, the specificity and accuracy increased to 91.6% and 91.3%, respectively, while the sensitivity remained at ~ 90%. Importantly, the FDR dropped from 57.3 to 30.8% (Fig. 4c; Additional file 2: Table S1D), suggesting that the high observed FDR was partially caused by misclassification of LTR sequences as TIR elements. We also removed LTR sequences from the TIRvish identified candidates and observed a 27% increase of specificity (80.5%) without any loss of sensitivity (94.5%; Fig. 4c; Additional file 2: Table S1D), suggesting that LTR sequences were a common source of false positives during structural identification of TIR elements.

In summary, MITE-Hunter and TIR-Learner showed the best performance for structural identification of MITEs and TIR elements (Fig. 4b, c), respectively, when TIR-Learner results were filtered to control false discovery (Fig. 4c). RepeatModeler, Repbase, and P-MITE had high accuracy but low sensitivity (Fig. 4a, b) and could be used to supplement structural annotations of MITE and TIR elements.

### Comparison of *Helitron* annotators

*Helitrons* are a subclass of DNA transposons that lack terminal repeats and do not generate target site duplications when transposed due to their rolling-circle mechanism of transposition [62], making identification of these elements particularly challenging. We found only one structure-based software, HelitronScanner [18], that is available, is bug-free (no errors in our test), and produced *Helitron* predictions.

HelitronScanner produced 52 Mb of raw candidate sequences in rice (13.9% of the genome; Additional file 2: Table S2). Since *Helitrons* may capture DNA sequences when transposed, many non-*Helitron* TE sequences and even protein-coding sequences are present in the raw prediction. Nested insertions between different TE classes are also likely to be present in these initial candidate sequences. Using the curated library, we found that 1.8% of *Helitron* candidates consisted of non-LTR sequences (LINEs and SINEs); 21% were LTR sequences and 11% were TIR sequences. With no filter applied, these *Helitron* candidates would include all classes of TEs, resulting in a high false discovery rate (93.7%; Additional file 2: Table S1E) and low annotation performance (Fig. 4d). To control for false discovery, we filtered *Helitron* candidates that lacked the signature 5′-TC...CTRR-3′ (R = G or A) terminal sequence structure, as well as those not inserted into AT or TT target sites (Additional file 1) [63]. We also removed non-*Helitron* TE sequences in these candidates using the curated library. After applying these filters, both the specificity and accuracy improved to 86%, while sensitivity was maintained at 95% (Fig. 4d; Additional file 2: Table S1E).

Similar to TIR-Learner for TIR element identification, HelitronScanner identified most of the curated *Helitrons* in the curated library, and also many additional elements not contained in the library (Fig. 4d). We further filtered these candidates with the EDTA pipeline (see the “Methods” section) and annotated the rice genome. Our filters yielded annotated sequences covering 7.3% of the rice genome compared to only 3.6% annotated using the curated library (Additional file 3: Table S4). Evaluation of the 30-bp sequences of both terminals with 10-bp flanking sequences as sequence logos showed the AT or TT target sites we required in our filtering and also that these candidates clearly have the canonical terminal structure 5′-TC...CTRR-3′ (with 5′-TC...CTAG-3′ dominating) which is required by HelitronScanner (Additional file 3: Figure S1). These candidates were also located in relatively AT-rich regions with significantly higher AT content in the 5′ terminal (Additional file 3: Figure S1), consistent with previous observations by Yang and Bennetzen regarding target site preference [64]. We found enriched CG content at the 3′ terminals especially at the − 13 and − 14 positions, which could produce a hairpin loop, a canonical *Helitron* feature [18]. While these elements contain the terminal features of a *Helitron*, this does not necessarily confirm their validity as intact elements. Further confirmation of these results will require meticulous curation and intra-specific comparisons [18, 63].

### Comparison of resource consumption and usage

In this study, we benchmarked 25 TE annotation programs and three databases, while nine others were attempted with failure due to a variety of reasons including (1) lack of maintenance with unresolved program bugs, (2) outdated programs required by the software and a lack of alternatives, (3) required programs or databases that are not open-source, and (4) programs take too long to run. For programs that were run successfully, some were more challenging than others. One of the main obstacles was installation. We found compile-free and precompiled programs were the easiest to use, followed by those available via conda and bioconda [65].

In addition to benchmarking the quality of the output of each program, we also benchmarked the algorithmic efficiency of these TE annotation programs. Since these programs were executed in different high-performance computational platforms (Additional file 2: Table S2), algorithmic performance could be slightly variable. Overall, most programs completed within 24 h with an average of 5.5 h (Additional file 2: Table S2). Longer run time was not associated with higher performance in terms of the six analysis metrics, and for some programs would become a barrier for annotation of large genomes. Most programs were not memory intensive, with a minimum of 7.2 Mbyte (SINE-Finder), an average of 8.7 Gbyte, and a maximum of 76 Gbyte (the GRF-LTR_FINDER method; Additional file 2: Table S2). Approximately two-thirds of the programs can be multi-threaded. However, the average CPU usage of programs was not significantly correlated with run time (*r* = − 0.19, *p* = 0.26, *F* test), indicating run time is primarily determined by algorithmic efficiency.

### Construction and benchmarking of the EDTA pipeline

**Fig. 5:**
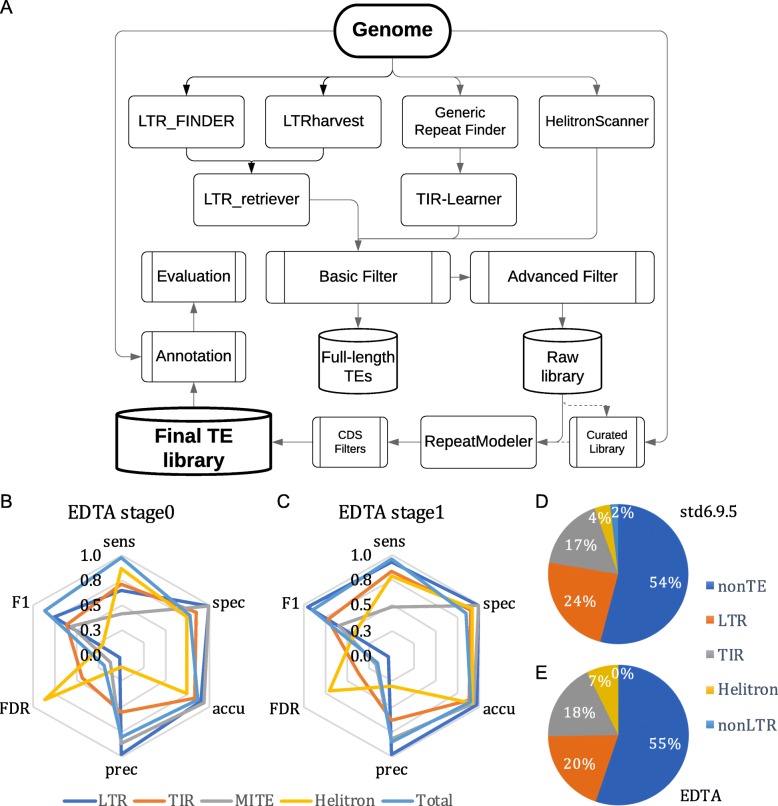
The Extensive *de-novo* TE Annotator (EDTA) pipeline. **a** The EDTA workflow. LTR retrotransposons, TIR elements, and *Helitron* candidates are identified from the genome sequence. Sublibraries (such as LTR library, TIR library, etc.) are filtered using EDTA library filtering scripts (including both basic filters and advanced filters, see the “Methods” section for details) for removal of misclassified TEs and are then used to mask TEs in the genome. The unmasked part of the genome is processed by RepeatModeler to identify non-LTR retrotransposons and any unclassified TEs that are missed by the structure-based library. Nested insertions and protein-coding sequences are removed in the final step to generate the final TE library. Performance of **b** EDTA stage 0 sublibraries and **c** EDTA stage 1 sublibraries after basic filtering and advanced filtering, respectively. Annotation of the rice genome using **d** the curated library and **e** the final EDTA-generated library

From the benchmarking results, we identified a set of programs that presented high sensitivity, specificity, and accuracy, but, in some instances, high FDR. Using these programs, we have developed a pipeline called Extensive *de-novo* TE Annotator (EDTA), which combines the best-performing programs and subsequent filtering methods for de novo identification of each TE subclass and compiles the results into a comprehensive non-redundant TE library. The EDTA pipeline incorporates LTRharvest, the parallel version of LTR_FINDER, LTR_retriever, GRF, TIR-Learner, HelitronScanner, and RepeatModeler as well as customized filtering scripts (Fig. 5a). We applied basic filters for LTR candidates, TIR candidates, *Helitron* candidates, and RepeatModeler results to remove short sequences, tandem repeats, and a portion of false positives (stage 0; the “Methods” section). Advanced filters were applied reciprocally for stage 0 sublibraries to further remove misclassified sequences (stage 1; the “Methods” section).

To test the performance of the EDTA pipeline, we annotated the rice genome using the curated TE library and the test library generated from the EDTA pipeline. Performance metrics for annotation generated using the stage 0 library showed low sensitivity (≤ 71%) for the annotation of LTR elements, TIR elements, and MITEs, and also suboptimal specificity (~ 75%) and accuracy (~ 76%) for *Helitron* annotations (Fig. 5b; Additional file 2: Table S1F). This is due to the nested TEs, captured TEs, or false discovery in *Helitron* candidates that impair the annotation performance in the combined stage 0 library. After reciprocal removal of misclassified TEs in each category (stage 1; Fig. 5a; the “Methods” section), the performance metrics were high for the EDTA stage 1 annotation (Fig. 5c). For all four TE subclasses and the overall repetitive sequences, the annotation sensitivity averaged 75.4%, specificity averaged 95.0%, and accuracy averaged 93.0% (Additional file 2: Table S1F). FDRs of these categories ranged from 3–36%, with the exception of *Helitrons* that had 70% of annotations not identified by the curated library (Additional file 2: Table S1F).

Overall, 96% of TEs were annotated in the rice genome using EDTA (Additional file 2: Table S1F), which was very close to the estimation based on the curated library (Fig. 5d, e). We did not identify any non-LTR retrotransposons with the RepeatModeler module (Fig. 5e). This is likely due to the low level of non-LTR elements in the rice genome (Table 1; Fig. 5d) that could have been misclassified as other TE subclasses, which is not the case for many of the larger eukaryotic genomes. Further annotation of non-LTR retrotransposons is necessary to exhaustively annotate TEs in the genome. As new programs become available for non-LTR elements, they will be benchmarked and potentially added to the EDTA pipeline based on performance metrics.

**Fig. 6:**
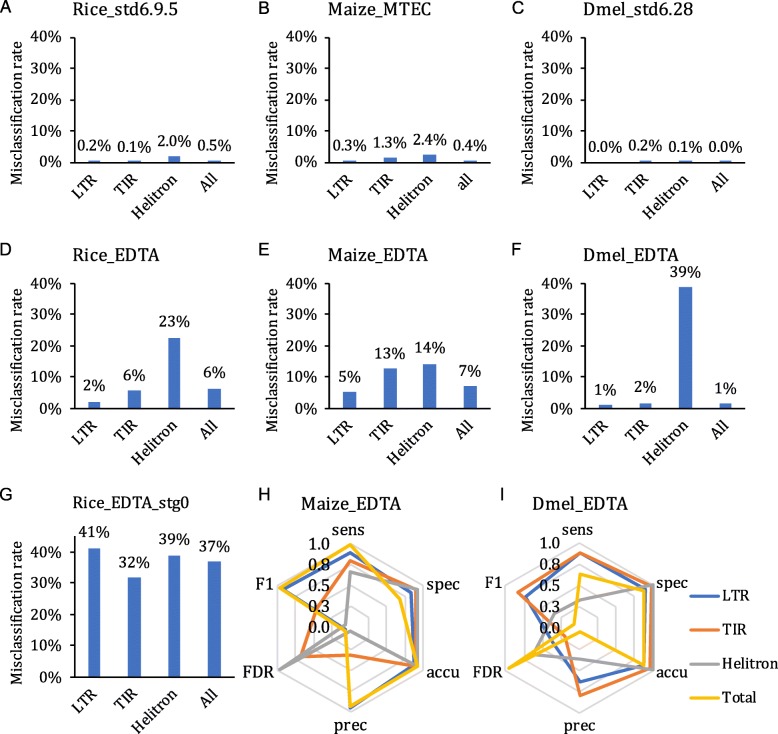
Benchmarking of the EDTA pipeline. Misclassification rate of whole-genome TEs annotated by **a** our curated rice library, **b** the Maize TE Consortium curated maize library (Maize_MTEC), **c** the community curated Drosophila library (Dmel_std6.28), **d** the EDTA-generated rice library, **e** the EDTA-generated maize library, **f** the EDTA-generated Drosophila library, and **g** the EDTA-generated stage 0 library with only basic filtering. Benchmarking of EDTA-generated maize (**h**) and Drosophila (**i**) libraries using Maize_MTEC and Dmel_std6.28 libraries, respectively

The purpose of EDTA is to ease the construction of non-redundant TE libraries for newly sequenced eukaryotic genomes, which can be subsequently used to generate whole-genome de novo TE annotations of structurally intact and fragmented elements. Our initial benchmarking was completed using the model species rice. To demonstrate its utility in other species, we applied the pipeline to maize [4, 66] and Drosophila [20], both of which have high-quality genomes and manually curated TE libraries to which we could compare the output of EDTA (Additional file 3: Tables S5-S6). Our results show that EDTA has high performance in the genomes of maize and Drosophila similar to that in the rice genome (Fig. 5c; Fig. 6h, i). Across the different types of TEs and species, sensitivity is averaged 77%, specificity is averaged 90%, and accuracy is averaged 92% (Fig. 6h, i; Additional file 2: Table S1F). EDTA annotated many more *Helitrons* in both species compared to their respective, curated libraries (FDR averaged 80%; Fig. 6h, i; Additional file 2: Table S1F), which is likely due to the incompleteness of curated libraries. In particular, the curated Drosophila library has only one *Helitron* sequence and this does not carry the canonical 5′-TC...CTRR-3′ terminal structure which is currently critical for automated identification of *Helitrons*.

We also estimated whole-genome TE misclassification rates for annotations generated by both the curated libraries and EDTA-generated libraries within each of the three species. Here, we define misclassification as TE sequences that are inconsistently classified into different subclasses (LTR retrotransposon, TIR transposon, and *Helitron*) in the whole-genome annotation (Additional file 1). The curated libraries demonstrate extremely low misclassification rate (≤ 2.4%) in each of the TE categories as well as the overall TE annotation (Fig. 6a–c), suggesting they are high quality with regard to classification consistency. This is expected based on the extensive manual curation efforts that have been employed to generate these curated libraries. To test the EDTA pipeline for this misclassification metric, we first evaluated annotations based on the best-performing structure-based programs without advanced downstream processing in rice. With only basic filtering (EDTA stage 0), the misclassification rate across TE subclasses ranged from 32 to 41% (Fig. 6g). However, the EDTA pipeline is more than just a compilation of programs, it also incorporates advanced filtering schemes to reduce misclassification and false identification of elements (Fig. 5a). Using the complete utility of the EDTA pipeline, which includes post hoc filtering scripts, the overall misclassification rate decreased to only 1–7% (Fig. 6d–f). Across the different classes of elements, the LTR and TIR elements in the three species were consistently classified across all of the species, with 1–13% of misclassification (Fig. 6d–f). *Helitrons* had a higher misclassification rate (14–39%), which is likely due to the sequence capture nature of these TEs. Our results indicate the potential need for careful verification and curation of *Helitron* annotations generated by HelitronScanner used within the EDTA pipeline.

There are a number of existing annotation pipelines for de novo TE annotation. REPET [67] is a well-known pipeline developed for de novo TE identification and annotation facilitated by the RepBase database [30]. Tephra was originally developed for structure-based TE annotations of the sunflower (*Helianthus annuus*) genome [68]. We benchmarked EDTA against these two pipelines in the rice genome. The overall sensitivity of these two pipelines (≥ 96%) was comparable to the EDTA result (96%), despite the lack of functionality of *Helitron* detection in REPET (Additional file 3: Figure S2; Additional file 2: Table S1F). However, neither of the programs were sensitive in TIR and MITE detection (27–62%), nor were they specific in LTR and *Helitron* (only for tephra) detection (67–78%; Additional file 3: Figure S2; Additional file 2: Table S1F). This suggests a poor balance between sensitivity and specificity. We also observed a high misclassification rate of the REPET annotation (15–62%) and a medium level of misclassification of the tephra annotation (21–36%; Additional file 3: Figure S2). The overall misclassification rate of REPET and tephra (22–28%; Additional file 3: Figure S2) is lower than that of the EDTA stage 0 annotation (37%; Fig. 6g), but much higher than the final EDTA annotation (6%; Fig. 6d).

Overall, we observed high performance of EDTA across multiple species including both plant and animal species with varying genome size and TE content. EDTA has demonstrated promise in automated high-quality TE annotations that facilitate whole-genome annotation and TE studies without the need for extensive manual annotation.

## Discussion

Recent innovations in third-generation (i.e., long-read) sequencing have enabled rapid and high-quality assembly of the repetitive fraction of genomes, creating an opportunity and need for high-throughput annotation of TEs. Annotation of TEs presents a substantial algorithmic and computational challenge. Different classes of TEs have distinct sequence characteristics, which has led to the development of software programs for each type. While anecdotally researchers have known the strengths and weaknesses of each of these methods, no comprehensive benchmarking study has quantified their relative annotation (i.e., sensitivity and specificity) and computational (i.e., run time and memory requirements) metrics. We have exhaustively tested these programs against a high-quality, manually curated rice TE library and have compiled the best-performing software as part of a comprehensive TE annotation pipeline known as EDTA. We have further demonstrated that the pipeline is robust across species.

All TEs were capable of transposition in the genome. However, the ability to amplify varies dramatically among different TE families. In fact, only a few TE families can amplify to high copy number. For example, in maize, the top 20 families of LTR retrotransposons comprise ~ 70% of the genome, whereas the remainder (380 or more) comprise only ~ 5% [69]. From this perspective, if a TE identification program captures elements with high copy number, the majority of the TE body in the genome will be characterized. Consistent with this notion, we observed that all general repeat identification programs, which depend on sequence repeatedness, performed well (high sensitivity and specificity, good precision and accuracy; Fig. 2a). Most importantly, the results from these programs are associated with very low FDR, suggesting when a sequence is repetitive to a certain degree, it is very likely to be a TE. However, most repeats from general programs are not classified and their sequence boundaries are often approximate. Not all tasks require TE classifications. For example, repetitive sequences are usually masked prior to gene annotation to minimize interference. For such purposes, general repeat identification programs and subsequent filtering for duplicated genes would suffice.

In contrast to the general repeat annotators, structure-based programs can identify low- or even single-copy elements and are therefore more sensitive. Moreover, these programs provide the exact coordinates of elements and are ideal for targeted study of TEs and their interactions with other components in the genome. However, based on our results, the majority of structure-based programs are associated with high FDR (up to 95%), and such error could be propagated in subsequent analyses. One factor contributing to this high error rate is misidentification due to nested insertion of TEs from different classes. We have developed an approach to minimize this issue by cross-checking sequences derived from programs for different classes of TEs. Another potential strategy to reduce FDR is to incorporate copy number control, but this would actually compromise the most important advantage of structure-based programs, which is sensitivity. Thus, this is an unsolvable problem without improvement to structure-based programs; particularly those for non-LTR retrotransposons and *Helitrons*. While more specific search engines or efficient filters may reduce the FDR, some level of manual curation may still be necessary for the generation of high-quality libraries.

Few species beyond rice have TE libraries of sufficient quality and genomes that are tractable enough to be used for benchmarking purposes. Furthermore, TEs comprise a relatively high proportion of the rice genome (~ 46%), and extensive manual curation efforts make it one of the only species in which a benchmarking study can reliably calculate true positive, false positive, true negative, and false negative rates across annotation programs. However, relative performance of TE annotation programs should be similar across systems. Programs have primarily been developed to detect specific types of TEs and are largely agnostic to species. This is possible because classes of TEs generally have similar structures across species [14, 16, 18]. Throughout this benchmarking exercise, we have based our tuning of programs (i.e., our educated parameters) on current knowledge of the structure of each target TE subclass [14, 16, 18], which, again, is not specialized to a particular system or species. As an example of the broad utility of these methods, the LTR_retriever program [40] has been tested for annotation of Arabidopsis, rice, maize, and sacred lotus (*Nelumbo nucifera*) [70] and demonstrated similar performance across systems. Furthermore, when we applied the EDTA pipeline to generate de novo libraries for maize and Drosophila, we saw consistent performance metrics to those observed for the initial benchmarking with rice.

We do anticipate some limits to the broad applicability of the EDTA pipeline across systems. For instance, based on our metrics, the performance of methods for detecting the non-LTR elements (i.e., SINEs and LINEs) was generally suboptimal and better algorithms are needed. Particularly, there is no structure-based program available for the identification of LINEs. The EDTA package may therefore miss a number of elements in, for instance, vertebrate genomes that contain many SINEs and LINEs [71]. Finally, our knowledge of TE structure is rapidly expanding, and parameterization and tuning of methods will therefore need to be continually updated. For example, variation in terminal motifs and target site duplication in LTR elements was previously poorly characterized. In the development of LTR_retriever, it was found that the terminal motif 5′-TG..CA-3′ occurs 99% of the time and that the vast majority of LTR TSDs are 5 bp [40]. While some programs set very flexible parameters for these features (e.g., LTRharvest), in our implementation of LTR_retriever, we applied our new knowledge and observed a substantial improvement in performance with regard to the FDR [40].

Moving forward, we see opportunities for improved annotation of highly variable TE classes including MITE/TIR elements and SINE/LINE, where, upon insertion, mutations and indels can be created. In these situations, construction of a consensus sequence is necessary for more precise TE annotation. Many programs do not currently have this feature. The GRF program for detection of interspersed repeats (*grf-intersperse*) has a consensus function, but the program does not allow indels, resulting in the lowest sensitivity but also the lowest FDR. For SINE/LINE detection, we found very low sensitivity and very high FDR, which is likely due to variation in these TEs (e.g., most LINEs are truncated upon insertion) and the lack of terminal repeats, making detection very challenging. Further development of consensus-based methods will be important. As new methods are generated and existing methods are improved, they will be benchmarked relative to our rice library and included in the EDTA pipeline when they result in a marked increase in annotation performance.

## Conclusions

Advances in sequencing technology are facilitating assembly of the repetitive portion of many genomes, which necessitates the annotation of these features. Using a highly curated library of rice TEs, we have created a benchmarking platform to test TE annotation software. We used this platform to exhaustively test currently available software based on output (i.e., sensitivity and specificity) as well as the performance of the software (i.e., run time and memory usage). From this benchmarking exercise, the EDTA pipeline was developed that combines the highest performing software with necessary filtering and processing scripts such that the pipeline can be applied to any new genome assembly.

## Methods

### Manual curation of transposable elements in rice

Manual curation of TEs in rice was started after the release of the map-based rice genome [22]. Repetitive sequences in the rice genome were compiled by RECON [44] with a copy number cutoff of 10. Details for manual curation of LTR sequences were previously described in the LTR_retriever paper [40]. In brief, for the curation of LTR retrotransposons, we first collected known LTR elements and used them to mask LTR candidates. Unmasked candidates were manually checked for terminal motifs, TSD sequences, and conserved coding sequences. Terminal repeats were aligned with extended sequences, from which candidates were discarded if alignments extended beyond their boundaries. For the curation of non-LTR retrotransposons, new candidates were required to have a poly-A tail and TSD. We also collected 13 curated SINE elements from [53] to complement our library.

For curation of DNA TEs with TIRs, flanking sequences (100 bp or longer, if necessary) were extracted and aligned using DIALIGN2 [72] to determine element boundaries. A boundary was defined as the position to which sequence homology is conserved over more than half of the aligned sequences. Then, sequences with defined boundaries were manually examined for the presence of TSD. To classify the TEs into families, features in the terminal and TSD sequences were used. Each transposon family is associated with distinct features in their terminal sequences and TSDs, which can be used to identify and classify elements into their respective families [14]. For *Helitron*s, each representative sequence requires at least two copies with intact terminal sequences, distinct flanking sequences, and inserts into “AT” target sites.

To make our non-redundant curated library, each new TE candidate was first masked by the current library. The unmasked candidates were further checked for structural integrity and conserved domains. For candidates that were partially masked and presented as true elements, the “80-80-80” rule (≥ 80% of the query aligned with ≥ 80% of identity and the alignment is ≥ 80 bp long) was applied to determine whether this element would be retained. For elements containing detectable known nested insertions, the nested portions were removed and the remaining regions were joined as a sequence. Finally, protein-coding sequences were removed using the ProtExcluder package [73]. The curated library version 6.9.5 was used in this study and is available as part of the EDTA toolkit.

### Calculation of benchmarking metrics

The curated TE annotation of the rice genome (*Oryza sativa* L. ssp. *japonica* cv. “Nipponbare” v. MSU7) was created using the standard library (v6.9.5) and RepeatMasker v4.0.8 with parameters “-pa 36 -q -no_is -norna -nolow -div 40 -cutoff 225.” These parameters identified homologous sequences with up to 40% divergence without detecting bacterial insertion elements, small RNA (pseudo) genes, and low complexity DNA. This annotation was used as the curated annotation for the calculation of benchmarking metrics. For genomic regions that cover more than 80% of a TE sequence in the curated library, the region was counted as a complete copy, and those that covered less than 80% were counted as a fragmented copy.

When we obtained a non-redundant test library from a target program (details in the next section), the test library was used to annotate the rice genome with the same RepeatMasker parameters, except that the test library was provided as a custom library. Then, the testing annotation was compared to the curated annotation for calculations of sensitivity, specificity, accuracy, precision, FDR, and *F*_1_ measures (Fig. 1). These six metrics were calculated using the script “lib-test.pl” in our EDTA toolkit.

### Execution of TE programs

We exhaustively searched the literature for open-source programs and databases that have been developed for both general repeat annotation and structural annotation. We executed each of these programs to obtain candidate sequences or downloaded sequences from specialized databases. All programs were executed using parameters consistent with current knowledge of TE structure (educated parameters). A description of each of these programs, observations we made about accessibility/ease of use of these programs, and the specific parameter options that were used are provided in Additional file 1. To benchmark the algorithmic efficiency, these programs were executed in multiple high-performance computing platforms (Additional file 2: Table S2). Run time (wall clock), average CPU usage, and maximum memory consumption were recorded using “/usr/bin/time -v.”

After we obtained raw sequences from programs, we went through three steps to construct non-redundant test libraries. The first step was to remove short tandem repeat contamination sequences that were present in the raw candidates. Identification of tandem sequences was achieved by Tandem Repeats Finder [74] with parameters “2 7 7 80 10 3000 2000 -ngs -h -l 6”. The second step was to remove missing characters (Ns) in candidates as well as short sequences. The minimum sequence length was set to 80 bp for TIR candidates and 100 bp for other types of TE candidates. We used the script “cleanup_tandem.pl” in the LTR_retriever package [40] for the first two steps with parameters “-misschar N -nc 50000 -nr 0.9 -minlen 100 (or 80) -minscore 3000 -trf 1 -cleanN 1.” The third step was to remove redundant sequences and nested insertions, which was achieved using the script “cleanup_nested.pl” in the LTR_retriever package [40] with default parameters. The third step was iterated five times to resolve heavily nested TEs for a thorough reduction of sequence redundancy. The resulting sequences were used as the non-redundant test library for the focal programs. Databases were used directly as test libraries without any filtering or manipulations.

### Construction of the Extensive *de-novo* TE annotator pipeline

Extensive *de-novo* TE Annotator (EDTA) is a pipeline for comprehensive and high-quality TE annotation for newly assembled eukaryotic genomes or to expand curated TE libraries. We combined open-source programs that are either specialized for a particular subclass of TEs or general for all repetitive sequences. The programs we selected had the highest performance from our benchmarking and together deliver the best TE annotation for a new genome that is possible given current program performance. Still, based on our benchmarking results, substantial contamination will exist due to misclassification of elements, nested insertions, and sequences captured by TEs.

The EDTA pipeline contains a set of scripts for filtering the output of each program to reduce the overall false discovery rate. The first set of scripts included in EDTA applies a simple filter for each of the initial predictions to remove tandem repeats and short sequences (< 80 bp for TIR elements and < 100 bp for LTR elements and *Helitrons*). For LTR candidates identified by LTRharvest and LTR_FINDER, false discoveries are filtered by LTR_retriever. For TIR candidates identified by TIR-Learner, sequences are reclassified as MITEs if their length is ≤ 600 bp. For *Helitron* candidates reported by HelitronScanner, filters based on target site (AT or TT) and prediction scores (≥ 12) are performed (Additional file 1).

To obtain high-quality intact TEs, higher level filters are applied to remove false positives. Terminal features of TIR elements and *Helitrons* are relatively short, which can cause them to be falsely reported based on the sequence of other TEs. In this case, the flanking sequence of these false elements is likely to have high copy number similar to their terminal sequences. To identify this source of false positives, the EDTA pipeline extracts 60-bp sequences centered on the start and end of candidate elements and searches for their copy number in the genome. Candidates with abundant full-length copies (≥ 20) in either terminus are determined to be false positives. For those with abundant full-length copies in both termini, a 60-bp sequence centered on the target site (30 bp joined from both flanking regions) is searched in the genome. If the copy number of both terminal regions are not significantly more (< 20,000 times) than that of the target site, the focal candidate is determined as a true candidate that is nested within the annotated element. After the above filtering, the EDTA pipeline uses *mdust* (© Dana-Farber Cancer Institute) to identify simple sequence repeat (SSR) in the remaining TIR and *Helitron* candidates. Elements carrying significant SSR sequences in either terminus (more than 15 out of 20 bp) are classified as false elements. SSR sequences are subsequently removed from any retained elements in the library. For LTR elements, due to the rigorous filtering and high-quality results produced by LTR_retriever, the list of intact LTR elements is reported as intact LTR elements. After these basic filtering steps, TE candidates are named stage 0 (full-length TEs in Fig. 5).

Advanced filters are necessary to generate a comprehensive and high-quality TE library. In stage 0 TE candidates, a fraction (0.3–27%) of them still contain misclassified TE sequences that are augmented when the library is used for whole-genome TE annotation. To further reduce misclassifications, TE sequences are filtered based on their relative richness between sublibraries. For each candidate sequence, the richness was estimated in both the target sublibrary (e.g., LTR) and the other sublibraries (e.g., TIR and *Helitron*) based on sequence homology. If the richness of the candidate sequence is not significantly higher in the target sublibrary than in another sublibrary, it is classified as a contaminant to the target sublibrary and discarded. Purification of TE candidates is performed reciprocally between sublibraries.

After these reciprocal filtering steps, updated sublibraries are aggregated and subjected to nested insertion removal and clustering, which generates the non-redundant stage 1 library (raw library in Fig. 5). Because LTR_retriever serves as a strong filter of results from LTRharvest and LTR_FINDER, no further filtering was necessary (LTR.stage0 = LTR.stage1). Non-redundant stage 1 TEs are then used to mask the genome. The remaining unmasked portion of the genome is scanned by RepeatModeler with default parameters to identify non-LTR retrotransposons and any unclassified TEs that are missed by structure-based TE identification. Finally, all remaining TEs are aggregated and protein-coding sequences are filtered in order to produce the final EDTA TE library. In this process, users can (1) provide TE-free coding sequences (CDS) of this species or closely related species for removal of gene-related sequences in the TE library and (2) provide a curated library; then, EDTA will only identify novel TEs that are not present in the provided library. All EDTA results presented here for rice and maize were based on de novo TE scans without using existing TE libraries. The EDTA library has RepeatMasker-readable sequence names and can be used to annotate whole-genome TE sequences.

To facilitate genome annotation and TE studies, we also provide a number of helpful functions in the EDTA package: (1) Users can white-list genomic regions from repeat masking (such as predicted gene regions); (2) output intact TE structural annotation information; (3) users can choose to annotate whole-genome TEs as well as perform low-threshold TE masking for downstream gene annotation; and (4) users can evaluate the TE annotation consistency without using external curated TE libraries.

## Supplementary information

**Additional file 1.** Supplementary Methods.

**Additional file 2: Table S1.** Performance metrics of TE methods. **Table S2.** Time and resource consumption.

**Additional file 3: Table S3.** Verification of new TIR candidates identified by TIR-Learner and MITE programs. **Table S4.** Comparison of whole-genome *Helitron* annotations using the curated library (v6.9.5), the HelitronScanner clean library (HS_clean), and the EDTA filtered HelitronScanner library (HS_EDTA). **Table S5.** TE content in the maize (*Zea mays* cv. ‘B73’ v. 4) genome. **Table S6.** TE content in the Drosophila (*Drosophila melanogaster* r6.28) genome. **Figure S1.** Sequence logos of terminal and flanking sequences of *Helitron* candidates cleaned by the standard library. **Figure S2.** Performance of TE annotation programs.

**Additional file 4.** Review history.

## Data Availability

The curated rice library and all scripts are freely available at https://github.com/oushujun/EDTA [34].

## References

[1] McClintock B (1947). Cytogenetic studies of maize and Neurospora. Year B Carnegie Inst Wash..

[2] Mills RE, Bennett EA, Iskow RC, Devine SE (2007). Which transposable elements are active in the human genome?. Trends Genet..

[3] Appels R, Eversole K, Feuillet C, Keller B, International Wheat Genome Sequencing Consortium (IWGSC), IWGSC RefSeq principal investigators (2018). Shifting the limits in wheat research and breeding using a fully annotated reference genome. Science.

[4] Schnable PS, Ware D, Fulton RS, Stein JC, Wei F, Pasternak S (2009). The B73 maize genome: complexity, diversity, and dynamics. Science..

[5] Marand AP, Zhao H, Zhang W, Zeng Z, Fang C, Jiang J (2019). Historical meiotic crossover hotspots fueled patterns of evolutionary divergence in rice. Plant Cell..

[6] Studer A, Zhao Q, Ross-Ibarra J, Doebley J (2011). Identification of a functional transposon insertion in the maize domestication gene *tb1*. Nat Genet..

[7] Huang C, Sun H, Xu D, Chen Q, Liang Y, Wang X (2018). *ZmCCT9* enhances maize adaptation to higher latitudes. Proc Natl Acad Sci U S A..

[8] Ou S, Chen J, Jiang N (2018). Assessing genome assembly quality using the LTR Assembly Index (LAI). Nucleic Acids Res..

[9] Campbell MS, Holt C, Moore B, Yandell M (2014). Genome annotation and curation using MAKER and MAKER-P. Curr Protoc Bioinformatics..

[10] Hoff KJ, Lange S, Lomsadze A, Borodovsky M, Stanke M (2016). BRAKER1: unsupervised RNA-Seq-Based genome annotation with GeneMark-ET and AUGUSTUS. Bioinformatics..

[11] Holt C, Yandell M (2011). MAKER2: an annotation pipeline and genome-database management tool for second-generation genome projects. BMC Bioinformatics..

[12] Kumar A, Bennetzen JL (1999). Plant retrotransposons. Annu Rev Genet..

[13] Eickbush TH, Jamburuthugoda VK (2008). The diversity of retrotransposons and the properties of their reverse transcriptases. Virus Res..

[14] Wicker T, Sabot F, Hua-Van A, Bennetzen JL, Capy P, Chalhoub B (2007). A unified classification system for eukaryotic transposable elements. Nat Rev Genet..

[15] Kunze R, Saedler H, Lönnig WE. Plant transposable elements. Adv Bot Res. 1997;27:331–470.

[16] Zhao D, Ferguson AA, Jiang N (1859). What makes up plant genomes: the vanishing line between transposable elements and genes. Biochim Biophys Acta..

[17] Su W, Gu X, Peterson T (2019). TIR-Learner, a new ensemble method for TIR transposable element annotation, provides evidence for abundant new transposable elements in the maize genome. Mol Plant..

[18] Xiong W, He L, Lai J, Dooner HK, Du C (2014). HelitronScanner uncovers a large overlooked cache of *Helitron* transposons in many plant genomes. Proc Natl Acad Sci U S A..

[19] Yang L, Bennetzen JL (2009). Distribution, diversity, evolution, and survival of *Helitrons* in the maize genome. Proc Natl Acad Sci U S A..

[20] Adams MD, Celniker SE, Holt RA, Evans CA, Gocayne JD, Amanatides PG (2000). The genome sequence of *Drosophila melanogaster*. Science..

[21] Initiative TAG. The Arabidopsis Genome Initiative. Analysis of the genome sequence of the flowering plant *Arabidopsis thaliana*. Nature. 2000;408:796–815. 10.1038/35048692 11130711

[22] Sasaki Takuji (2005). The map-based sequence of the rice genome. Nature.

[23] Copetti D, Zhang J, El Baidouri M, Gao D, Wang J, Barghini E (2015). RiTE database: a resource database for genus-wide rice genomics and evolutionary biology. BMC Genomics..

[24] Goerner-Potvin P, Bourque G (2018). Computational tools to unmask transposable elements. Nat Rev Genet..

[25] Lerat E (2010). Identifying repeats and transposable elements in sequenced genomes: how to find your way through the dense forest of programs. Heredity.

[26] Price AL, Jones NC, Pevzner PA (2005). *De novo* identification of repeat families in large genomes. Bioinformatics..

[27] Girgis HZ (2015). Red: an intelligent, rapid, accurate tool for detecting repeats *de-novo* on the genomic scale. BMC Bioinformatics..

[28] Smit AFA, Hubley R (2015). RepeatModeler Open-1.0. 2008—2015.

[29] Smit AFA, Hubley R, Green P (2015). RepeatMasker Open-4.0. 2013--2015.

[30] Bao W, Kojima KK, Kohany O (2015). Repbase Update, a database of repetitive elements in eukaryotic genomes. Mob DNA..

[31] Chen J, Hu Q, Zhang Y, Lu C, Kuang H (2014). P-MITE: a database for plant miniature inverted-repeat transposable elements. Nucleic Acids Res..

[32] Vassetzky NS, Kramerov DA (2013). SINEBase: a database and tool for SINE analysis. Nucleic Acids Res..

[33] Hoen DR, Hickey G, Bourque G, Casacuberta J, Cordaux R, Feschotte C (2015). A call for benchmarking transposable element annotation methods. Mob DNA..

[34] Ou S, Su W. The Extensive de-novo TE Annotator. GitHub. Available from: https://github.com/oushujun/EDTA. Accessed 15 Nov 2019.

[35] Jiang N, Bao Z, Zhang X, Hirochika H, Eddy SR, McCouch SR (2003). An active DNA transposon family in rice. Nature..

[36] Jiang N, Bao Z, Zhang X, Eddy SR, Wessler SR (2004). Pack-MULE transposable elements mediate gene evolution in plants. Nature..

[37] Feschotte C, Swamy L, Wessler SR (2003). Genome-wide analysis of *mariner*-like transposable elements in rice reveals complex relationships with stowaway miniature inverted repeat transposable elements (MITEs). Genetics..

[38] Xie Y, Wang Y, Wu R. A rice DNA sequence that resembles the maize *Mu 1* transposable element. Rice Genetics Collect. 2008;2:377–87.

[39] Barret P, Brinkman M, Beckert M (2006). A sequence related to rice *Pong* transposable element displays transcriptional activation by *in vitro* culture and reveals somaclonal variations in maize. Genome..

[40] Ou S, Jiang N (2018). LTR_retriever: a highly accurate and sensitive program for identification of long terminal repeat retrotransposons. Plant Physiol..

[41] Zhang X, Jiang N, Feschotte C, Wessler SR (2004). *PIF*- and *Pong*-like transposable elements: distribution, evolution and relationship with *Tourist*-like miniature inverted-repeat transposable elements. Genetics..

[42] Han Y, Qin S, Wessler SR (2013). Comparison of class 2 transposable elements at superfamily resolution reveals conserved and distinct features in cereal grass genomes. BMC Genomics..

[43] Chen J, Lu L, Benjamin J, Diaz S, Hancock CN, Stajich JE (2019). Tracking the origin of two genetic components associated with transposable element bursts in domesticated rice. Nat Commun..

[44] Bao Z, Eddy SR (2002). Automated *de novo* identification of repeat sequence families in sequenced genomes. Genome Res..

[45] Shi Jieming, Liang Chun (2019). Generic Repeat Finder: A High-Sensitivity Tool for Genome-Wide De Novo Repeat Detection. Plant Physiology.

[46] McCarthy EM, McDonald JF (2003). LTR_STRUC: a novel search and identification program for LTR retrotransposons. Bioinformatics..

[47] Xu Z, Wang H (2007). LTR_FINDER: an efficient tool for the prediction of full-length LTR retrotransposons. Nucleic Acids Res..

[48] Ellinghaus D, Kurtz S, Willhoeft U (2008). LTRharvest, an efficient and flexible software for *de novo* detection of LTR retrotransposons. BMC Bioinformatics..

[49] Lee H, Lee M, Mohammed Ismail W, Rho M, Fox GC, Oh S (2016). MGEScan: a Galaxy-based system for identifying retrotransposons in genomes. Bioinformatics..

[50] Valencia JD, Girgis HZ (2019). LtrDetector: a tool-suite for detecting long terminal repeat retrotransposons de-novo. BMC Genomics..

[51] Rho M, Choi J-H, Kim S, Lynch M, Tang H (2007). *De novo* identification of LTR retrotransposons in eukaryotic genomes. BMC Genomics..

[52] Ou S, Jiang N. LTR_FINDER_parallel: parallelization of LTR_FINDER enabling rapid identification of long terminal repeat retrotransposons. bioRxiv. 2019:722736 Available from: https://www.biorxiv.org/content/10.1101/722736v1. [cited 2019 Aug 17]. 10.1186/s13100-019-0193-0 PMC6909508 31857828

[53] Mao H, Wang H (2017). SINE_scan: an efficient tool to discover short interspersed nuclear elements (SINEs) in large-scale genomic datasets. Bioinformatics..

[54] Warburton PE, Giordano J, Cheung F, Gelfand Y, Benson G (2004). Inverted repeat structure of the human genome: the X-chromosome contains a preponderance of large, highly homologous inverted repeats that contain testes genes. Genome Res..

[55] Gremme G, Steinbiss S, Kurtz S (2013). GenomeTools: a comprehensive software library for efficient processing of structured genome annotations. IEEE/ACM Trans Comput Biol Bioinform..

[56] Han Y, Wessler SR (2010). MITE-Hunter: a program for discovering miniature inverted-repeat transposable elements from genomic sequences. Nucleic Acids Res..

[57] Ye C, Ji G, Liang C (2016). detectMITE: a novel approach to detect miniature inverted repeat transposable elements in genomes. Sci Rep.

[58] Ge R, Mai G, Zhang R, Wu X, Wu Q, Zhou F (2017). MUSTv2: an improved *de novo* detection program for recently active miniature inverted repeat transposable elements (MITEs). J Integr Bioinform..

[59] Hu J, Zheng Y, Shang X (2018). MiteFinderII: a novel tool to identify miniature inverted-repeat transposable elements hidden in eukaryotic genomes. BMC Med Genomics..

[60] Crescente JM, Zavallo D, Helguera M, Vanzetti LS (2018). MITE Tracker: an accurate approach to identify miniature inverted-repeat transposable elements in large genomes. BMC Bioinformatics..

[61] Du C, Hoffman A, He L, Caronna J, Dooner HK (2011). The complete *Ac*/*Ds* transposon family of maize. BMC Genomics..

[62] Kapitonov VV, Jurka J (2001). Rolling-circle transposons in eukaryotes. Proc Natl Acad Sci U S A..

[63] Thomas J, Pritham EJ (2015). *Helitrons*, the eukaryotic rolling-circle transposable elements. Microbiol Spectr..

[64] Yang L, Bennetzen JL (2009). Structure-based discovery and description of plant and animal *Helitrons*. Proc Natl Acad Sci U S A..

[65] Grüning B, Dale R, Sjödin A, Chapman BA, Rowe J, The Bioconda Team (2018). Bioconda: sustainable and comprehensive software distribution for the life sciences. Nature Methods..

[66] Jiao Y, Peluso P, Shi J, Liang T, Stitzer MC, Wang B (2017). Improved maize reference genome with single-molecule technologies. Nature..

[67] Flutre T, Duprat E, Feuillet C, Quesneville H (2011). Considering transposable element diversification in *de novo* annotation approaches. PLoS One..

[68] Badouin H, Gouzy J, Grassa CJ, Murat F, Staton SE, Cottret L (2017). The sunflower genome provides insights into oil metabolism, flowering and Asterid evolution. Nature..

[69] Baucom RS, Estill JC, Chaparro C, Upshaw N, Jogi A, Deragon J-M (2009). Exceptional diversity, non-random distribution, and rapid evolution of retroelements in the B73 maize genome. PLoS Genet..

[70] Ming R, VanBuren R, Liu Y, Yang M, Han Y, Li L-T (2013). Genome of the long-living sacred lotus (*Nelumbo nucifera* Gaertn.). Genome Biol.

[71] Kvikstad EM, Makova KD (2010). The (r)evolution of SINE versus LINE distributions in primate genomes: Sex chromosomes are important. Genome Res..

[72] Morgenstern B, Werner N, Prohaska SJ, Steinkamp R, Schneider I, Subramanian AR (2005). Multiple sequence alignment with user-defined constraints at GOBICS. Bioinformatics..

[73] Campbell MS, Law M, Holt C, Stein JC, Moghe GD, Hufnagel DE (2014). MAKER-P: a tool kit for the rapid creation, management, and quality control of plant genome annotations. Plant Physiol..

[74] Benson G (1999). Tandem repeats finder: a program to analyze DNA sequences. Nucleic Acids Res..

