# Supplementary material for: Benchmarking transposable element annotation methods for creation of a streamlined, comprehensive pipeline
Source: Genome Biol. 2019 Dec 16;20:275. doi: 10.1186/s13059-019-1905-y (PMC6913007; doi:10.1186/s13059-019-1905-y)
Supplement: Supplementary file 1 — Additional file 1. Supplementary Methods. [file 13059_2019_1905_MOESM1_ESM.docx]

## Additional File 1. Supplementary Methods

### Parameter settings of TE programs

Details regarding program parameters and user experience are described below. For processing of results, please refer to the Methods section of this study.

*General repeat annotators*

**RECON** [[1]](https://paperpile.com/c/Ovk0Hl/xhYU) was developed for *de novo* identification and classification of repeat sequences based on multiple sequence alignment information. We used the RepeatModeler patched version (v1.08) in our test. The initial all-vs-all pairwise comparison of the rice genome sequence was performed with BLAST (v2.2.31). Removal of self-alignments and formatting for RECON required using the script “Filter_and_format_blast_for_RECON.pl” in our Extensive *de-novo* TE Annotator (EDTA) toolkit. Because RECON is a single-threaded program, we ran RECON separately for each chromosome with default parameters, and combined the results afterwards.

**RepeatScout** [[2]](https://paperpile.com/c/Ovk0Hl/PObp) is a *k*-mer based program to identify repetitive sequences. The version 1.0.5 was used in this study, which is single-threaded. We used 16 bp as the starting seed length (-l 16) and minimum copy number of 10 (--thresh=10) for *de novo* repeat identification.

**RepeatModeler** [[3]](https://paperpile.com/c/Ovk0Hl/8ZJO) is a *de novo* repeat identification and classification program which was built based on RepeatScout [[2]](https://paperpile.com/c/Ovk0Hl/PObp) and RECON [[1]](https://paperpile.com/c/Ovk0Hl/xhYU). RepeatModeler helps to refine TE boundaries, classify TEs based on a genomic database, and construct non-redundant TE libraries for whole-genome annotation. We used the v1.0.11 version of RepeatModeler that was multithreading-enabled with the NCBI BLAST engine (-engine ncbi). We ran RepeatModeler with default parameters.

**Red** [[4]](https://paperpile.com/c/Ovk0Hl/1SNe) is a C++ program which enables rapid detection of repetitive sequences. The software is not dependent on other programs and is available in bioconda. Parameters for running Red are automatically defined based on the input genome sequence. Repeat candidates detected by Red are formatted as a list of genome coordinates; sequences were extracted using the “call_seq_by_list.pl” script in our EDTA toolkit.

**Generic Repeat Finder (GRF)** [[5]](https://paperpile.com/c/Ovk0Hl/ByTu) is a C++ program that can detect multiple types of repeats including interspersed repeats, terminal inverted repeats (TIR, including MITEs), and terminal direct repeats (TDR, including LTR retrotransposons). The grf-intersperse module of GRF was used to detect interspersed repeats in groups (-f 0 -c 5), and the grf-alignment2 module was further used to generate consensus sequences. The “get_interspersed_consensus.pl” script in our EDTA toolkit was developed to extract consensus sequences with filtering of the minimum sequence length of 80 bp. The CD-HIT [[6]](https://paperpile.com/c/Ovk0Hl/0a2b) program was used to further cluster consensus sequences with a minimum of 99% coverage and 80% identity (-c 0.80 -n 5 -d 0 -aL 0.99 -s 0.8 -M 0).

**RepBase** [[7]](https://paperpile.com/c/Ovk0Hl/w23c) is a database of consensus TE sequences derived from known eukaryotic genomes, which has been incorporated in RepeatMasker [[3]](https://paperpile.com/c/Ovk0Hl/8ZJO) for whole-genome TE annotations. We used the rice database in RepBase to benchmark its annotation performance by comparing to our curated database. The RepeatMasker version v4.0.8 and RepBase version 20170127 was used (-species rice -e Crossmatch). We also tested the non-rice database in RepBase for *de novo* TE annotation of rice. All core and comprehensive sequences that are not derived from rice were used in the test.

Other methods that were tried included CENSOR [[8]](https://paperpile.com/c/Ovk0Hl/PJZJ) and PILER [[9]](https://paperpile.com/c/Ovk0Hl/uMnX). CENSOR requires a .map file that was not described in the manual and on the website, thus we could not decipher its format and usage. CENSOR rely on RepBase libraries, which has been commercialized recently and requires membership for any use and is not compatible with the open-source toolkit we developed. PILER relies on whole-genome self-alignment for detection of TEs. The step uses PALS [[9]](https://paperpile.com/c/Ovk0Hl/uMnX), which has been deprecated.

*LTR retrotransposons*

**LTR_STRUC** [[10]](https://paperpile.com/c/Ovk0Hl/SxBQ) is one of the earliest programs developed for detection of long terminal repeat (LTR) retrotransposons. It is Windows-based and requires a non-commercial license. LTR_STRUC requires no parameter settings and generates four files for each candidate. The script “convert_ltr_struc.pl” in the LTR_retriever program [[11]](https://paperpile.com/c/Ovk0Hl/CT05) was used to extract coordinate information from LTR_STRUC outputs.

**LTR_FINDER** [[12]](https://paperpile.com/c/Ovk0Hl/nwsc) is a commonly used program for *de novo* identification of LTR retrotransposons. It allows many parameters to be set. However, LTR_FINDER only allows one-CPU for all jobs, limiting the scalability of this program. To work around this limitation, we developed a multithreading wrapper called “LTR_FINDER_parallel.pl” [[13]](https://paperpile.com/c/Ovk0Hl/bpra), which will chop the genome into small pieces to run LTR_FINDER in parallel. We used 1-Mb chunks and 300 timeout seconds for each chunk (-harvest_out -size 1000000 -time 300). LTR_FINDER version 1.0.6 was used with the minimum length of LTR regions, the maximum length of LTR regions, the maximum length of the whole candidate, and the maximum divergence between terminal repeats set to 100 bp, 7000 bp, 15,000 bp, and 85%, respectively (-w 2 -C -D 15000 -d 1000 -L 7000 -l 100 -p 20 -M 0.85). The script “convert_ltr_finder.pl” in the LTR_retriever program [[11]](https://paperpile.com/c/Ovk0Hl/CT05) was used to extract coordinate information from LTR_FINDER outputs.

**LTRharvest** [[14]](https://paperpile.com/c/Ovk0Hl/Ch9e) is another popular program for *de novo* LTR detection. The availability of precompiled binary code makes it user friendly. While LTRharvest runs on only 1 CPU, it has a relatively short run time. We used version 1.5.10 for our analyses, with parameters similar to what we applied in LTR_FINDER (-minlenltr 100 -maxlenltr 7000 -mintsd 4 -maxtsd 6 -similar 85 -vic 10 -seed 20 -seqids yes). We also specified the canonical terminal motif 5'-TG...CA-3' with a maximum of 1 bp mismatch for the detection of LTR candidates (-motif TGCA -motifmis 1).

**MGEScan3** [[15]](https://paperpile.com/c/Ovk0Hl/sED5) is a Galaxy-based program that combines MGEScan_LTR [[16]](https://paperpile.com/c/Ovk0Hl/I4QL) and MGEScan-non-LTR [[17]](https://paperpile.com/c/Ovk0Hl/ZPaW). Thus, it detects both LTR and non-LTR retrotransposons. The command line tool is easy to use, but does not allow specific parameters to be set for either algorithm. The program is multithreading-enabled and is relatively quick. The script “convert_MGEScan3.0.pl” in the LTR_retriever program [[11]](https://paperpile.com/c/Ovk0Hl/CT05) was used to extract coordinate information from MGEScan3 outputs.

**LtrDetector** [[18]](https://paperpile.com/c/Ovk0Hl/KQgL) is a recently developed tool for *de novo* LTR annotation. Installation of this C++ program was straight-forward, though it requires a very new compiler (gcc/8.2.0). The default options for the LtrDetector parameters were used. The default bed format output of LtrDetector includes target site duplication (TSD) as part of the candidate. We used the script “convert_ltrdetector.pl” in the LTR_retriever program [[11]](https://paperpile.com/c/Ovk0Hl/CT05) to remove TSD coordinates and extract LTR information for further analyses.

**GRF** [[5]](https://paperpile.com/c/Ovk0Hl/ByTu) can detect terminal direct repeats (TDR), which could be provided to a modified version of LTR_FINDER for detection of LTR retrotransposons. We first used our best practice parameters to obtain raw TDRs; however, the modified LTR_FINDER could not detect any LTR candidates. We then used the parameters provided in the manual (-c 2 --min_tr 100 --min_space 1090 --max_space 23490 --match 2 --mismatch 2 --indel 3 -f 1 -p 30) to run *grf-main*, and used *grf-filter* to filter out extreme TDRs (*grf-filter* 100 3500 1000 20000). Finally, the modified LTR_FINDER provided in the GRF package was executed following the options suggested in the program manual. Using these options, a large number of candidates were obtained, and coordinate information was extracted using the script “convert_ltr_finder.pl” in the LTR_retriever program [[11]](https://paperpile.com/c/Ovk0Hl/CT05) for further analysis.

**LTR_retriever** [[11]](https://paperpile.com/c/Ovk0Hl/CT05) is a Perl program that was designed to filter excessive false positives in *de novo* LTR detection and construct a high-quality non-redundant library for whole-genome annotation. Input files are very flexible for LTR_retriever; for example, it can utilize the output of LTR-related programs and produce high-quality LTR candidates. We ran LTR_retriever on outputs of LTR_STRUC, MGEScan3, LTR_FINDER, LTRharvest, LtrDetector, and GRF-LTR_FINDER, respectively, and also on all six program outputs combined. Since LTR_retriever has been optimized for the best balance between sensitivity and specificity, we used default parameters for all LTR_retriever analyses.

We also tried LTR_MINER [[19]](https://paperpile.com/c/Ovk0Hl/cN8z) and LTR Annotator [[20]](https://paperpile.com/c/Ovk0Hl/ECgZ). While LTR_MINER is a Perl program designed to annotate intact and solo LTR elements from RepeatMasker output files, we could not get it to work due to changes in the RepeatMasker output format in newer versions of the program. For LTR Annotator, we could not find any installation package associated with this name from both their paper and a Google search.

*nonLTR (SINE and LINE)*

**SINEBase** [[21]](https://paperpile.com/c/Ovk0Hl/tiOB) is a database of short interspersed nuclear elements (SINEs). We downloaded consensus sequences from <http://sines.eimb.ru/> in both SINEBank and LINEBank for a collection of SINE families and partner LINEs, respectively. A total of 228 elements were downloaded.

**MGEScan3** [[15]](https://paperpile.com/c/Ovk0Hl/sED5) was used to *de novo* detect non-LTR retrotransposons. There is no parameter settings for this program other than being multi-threadible. We found only a small number of candidates (n = 20) using MGEScan3 in the rice genome.

**SINE-Finder** [[22]](https://paperpile.com/c/Ovk0Hl/t2OV) is a Python program designed to identify tRNA-derived SINEs. There is no installation required for this program and parameter settings are straightforward. However, only the forward strand of the input sequences could be searched without errors, which requires manually generating and providing the reverse complement strand for the complete search. A total of 431 raw candidates were identified.

**SINE_Scan** [[23]](https://paperpile.com/c/Ovk0Hl/qQFq) is a Perl program representing the latest development of *de novo* SINE identification methods which is based on SINE-Finder. SINE_Scan can identify all three known types of SINEs, which are tRNA, 7SLRNA, and 5SRNA. SINE_Scan allows users to start their analyses from different steps and generates multi-sequence alignment (MSA) files for each candidate for manual curation. We used default parameters to run SINE_Scan and identified a total of 35 SINE family candidates in rice.

*TIR*

**P-MITE** [[24]](https://paperpile.com/c/Ovk0Hl/dsR1) is a database of plant Miniature Inverted Transposable Elements (MITEs), which contains 3,527 MITE families obtained from 41 plant species. Download links for the P-MITE database were broken (<http://pmite.hzau.edu.cn/download/>). We obtained non-redundant and TSD-removed MITE sequences from Dr. Jiongjiong Chen. We used rice and non-rice datasets to test the annotation performance of the P-MITE database.

**IRF** [[25]](https://paperpile.com/c/Ovk0Hl/5STs) is a multi-platform software designed to identify inverted repeat elements. It has five required parameters and 19 optional parameters. Some of the IRF parameters are quite technical for inexperienced users, such as matching/mismatching scores, indel penalty, and match/indel probabilities. We used parameters of matching weight +2, mismatch penalty -3, indel penalty -5, default match and indel probabilities 80 and 10, respectively, minimum score of 20, maximum stem length of 10000, maximum loop of 10000, and identity value of 80 ( “2 3 5 80 10 20 10000 10000 -h -r 80 -t5 10000”). The rice genome was split into 12 chromosomes, and each chromosome was processed separately. The output of IRF software is a .dat file, with coordinates and other information, therefore, users need to take one more step to obtain the FASTA file from the IRF output.

**MITE-Hunter** [[26]](https://paperpile.com/c/Ovk0Hl/Aa1t) is a Perl program and installation requires formatdb, blastall, mdust, and muscle as prerequisites. The software blastall and formatdb are outdated, and have been replaced by the software package BLAST+. MITE-Hunter has 17 parameter options and some are redundant. We used parameters of maximum unmatched 2 bp in TIR region, the maximum length of 1000 bp, a minimum shared length of 80 bp between TEs that will be grouped together, and minimum copy number of 1 ( “-l 2 -w 1000 -L 80 -m 1 -S 12345678 -c 16”). One of the best features for MITE-Hunter is that this software can take checkpoints. The -S parameter allows the user to start the program from a specific step. Therefore, if there is anything wrong in the running process, users do not have to restart from the beginning. MITE-Hunter generates hundreds of intermediate files in the same folder, some of which are very large. This could cause difficulties for users to obtain the final output and organize files in the target folder.

**TIRvish** is a program module in the GenomeTools [[27]](https://paperpile.com/c/Ovk0Hl/ZD7K) package. The binary version of this package makes it free of installation. The output of TIRvish is a GFF file with coordinates for the complete candidate element, the TIR regions of the element, and the TSDs of the element. We used parameters “-seed 20 -mintirlen 10 -maxtirlen 1000 -mintirdist 10 -maxtirdist 20000 -similar 80 -mintsd 2 -maxtsd 11 -vic 13 -seqids yes” to identify TIR candidates with TIRs ranging from 10 - 1000 bp with at least 80% of identity, TIR distance from 10 bp - 20 kb, and TSDs from 2 - 11 bp. TIRvish was the easiest and fastest TIR/MITE program we tested. However, the element boundaries defined by TIRvish are frequently shifted ± 1-2 bp, which can have major impacts on downstream analyses.

**detectMITE** [[28]](https://paperpile.com/c/Ovk0Hl/UxsV) is an open-source MATLAB program for *de novo* detection of MITEs. Its installation requires a third-party software CD-HIT [[6]](https://paperpile.com/c/Ovk0Hl/0a2b). The output of detectMITE is a FASTA file that includes coordinates, TIR length, and TSD length. We identified MITEs of maximum length 1000 bp (matlab -nodisplay -nosplash -r tic; do_MITE_detection('Rice.fa', '-mite_maximum_length',1000, '-genome','Rice_detectMITE', '-cpu',16); runtime=toc; quit).

**GRF** [[5]](https://paperpile.com/c/Ovk0Hl/ByTu) is an open-source C++ software on Github. GRF can find multiple types of repeats including terminal inverted repeats (TIRs) and MITEs. GRF requires the CD-HIT software [[6]](https://paperpile.com/c/Ovk0Hl/0a2b) for clustering of repeat candidates. Users can specify the length of TIR/TSD and also the minimum and/or the maximum length of the candidates. We used parameters “-c 0 -t 16 -p 20 --min_space 10 --max_space 10000 --max_indel 0 --min_tr 10 --min_spacer_len 10 --max_spacer_len 10000” for inverted repeats detection, and parameters “-c 1 -t 16 -p 20 --min_space 10 --max_space 10000 --max_indel 0 --min_tr 10 --min_spacer_len 10 --max_spacer_len 10000 --min_tsd 2 --max_tsd 10” for MITE detection. We obtained an unrealistically large number of candidates from both processes, which included 630 Gb and 47 Gb of raw candidates for *grf-main* and *grf-mite*, respectively. We also used default parameters for *grf-mite* to obtain 1.5 Mb of raw candidates. All these results were processed and presented in the benchmarking result (Supplementary Table S1) as GRF-TIR_edu, GRF-mite_edu, and GRF-mite_dft, respectively.

One of the main reasons for the large size of GRF-TIR_edu and GRF-mite_edu raw results is that the majority of these candidates are overlapping and nested within each other, and GRF failed to filter these low-quality candidates. We employed extensive filters to remove overlapping candidates based on the following rules: 1) the minimum length of candidates and their inverted repeat is set to 80 bp and 25 bp (combination of head and tail), respectively. 2) for GRF-mite_edu candidates, target site duplications (TSDs) are required to be present. 3) for candidates with shared start coordinates, retain the candidate with less SNPs and Indels in the inverted repeat region. If multiple start-shared candidates have the same number of mutations in their TIRs, the shortest candidate is retained. 4) for candidates with shared stop coordinates, similar filters were applied as described in (3). 5) for candidates with start coordinates that differ in +/- 1 bp, the candidate with the longer TSD is retained. These filtering steps were implemented in the script “clean_GRF_TIR.pl” in our EDTA toolkit. We also used the script “cleanup_tandem.pl” in our EDTA toolkit to remove tandem repeats in these candidates. After these filters, the size of candidates dropped down to 1.2 Gb and 1.6 Gb for GRF-TIR_edu and GRF-mite_edu, respectively. We further used the script “cleanup_nested.pl” in our EDTA toolkit to remove redundant sequences and nested insertions in the remaining candidates. This step was iterated five times for thorough reduction of redundancy. For MITE candidates, based on a somewhat arbitrary definition, their length should not exceed 600 bp [[29]](https://paperpile.com/c/Ovk0Hl/tWbe), we thus removed candidates that are longer than 600 bp. Finally, the cleaned and non-redundant test library for TIR and MITE are 304 Mb and 10 Mb, respectively.

**miteFinderII** [[30]](https://paperpile.com/c/Ovk0Hl/1Kjh) is an open source C++ software on Github. miteFinderII does not have as many parameter options as others, we used the default parameters (-threshold 0.5). miteFinderII has limited instructions in the README file and refers most questions to the publication.

**MITE-Tracker** [[31]](https://paperpile.com/c/Ovk0Hl/rf9h) is a Python3 package, which needs vsearch [[32]](https://paperpile.com/c/Ovk0Hl/eNIp) for the clustering process. MITE-tracker parameters include the minimum and the maximum length of MITE elements, as well as the minimum and the maximum length of TSD. We used parameters “-w 16 --tsd_min_len 2 --tsd_max_len 9 --mite_min_len 50 --mite_max_len 1000 --task candidates -j Rice” to obtain MITE candidates. Raw candidates contained many false positive sequences with the terminal inverted repeat structure, which may not be useful for TE annotation. The number of clustered MITE candidates is comparable to other programs.

**MUSTv2** [[33]](https://paperpile.com/c/Ovk0Hl/OUyw) is a Perl program that requires BLAST, BLAT [[34]](https://paperpile.com/c/Ovk0Hl/Wkpl), and several Perl packages such as Bioperl [[35]](https://paperpile.com/c/Ovk0Hl/M20w). We used default parameters. The output of MUSTv2 is a .txt file with MITE information such as ID, cluster, coordinates, strand, length, TIR, TSD, and scores. Users need to further process the output .txt file to get the FASTA file of candidate MITEs.

**TIR-Learner** [[36]](https://paperpile.com/c/Ovk0Hl/WdzE) is a Python3 program that uses machine-learning algorithms to facilitate identification and classification of TIR elements. TIR-Learner was originally designed for maize TIR element annotations using IRF [[25]](https://paperpile.com/c/Ovk0Hl/5STs) as the TIR structure search engine. In this study, we modified it for the rice genome by using the curated library to train the machine-learning classifier. In version 1.19 of TIR-Learner, GRF [[5]](https://paperpile.com/c/Ovk0Hl/ByTu) is used as the TIR search engine for faster performance compared to IRF. We also explored using TIRvish [[27]](https://paperpile.com/c/Ovk0Hl/ZD7K) as an even faster search engine, but had a much lower sensitivity compared to that of the GRF engine due to the imprecise candidate boundary defined by TIRvish, which result in decreased classification accuracy, and a high false negative rate for TIR-Learner.

TIR-Learner integrates the homology-based method and *de novo* machine learning-based method. We set the minimum TIR=10 and used different length and sequence motifs for identification of TSDs of different TIR superfamilies according to the literature [[37]](https://paperpile.com/c/Ovk0Hl/hMnn). The outputs of TIR-Learner include a gff3 file and a FASTA file. Each entry includes information such as coordinates, superfamily, TIR/TSD sequences/identity, and TE length. To remove potential contaminants from other types of TEs such as LTR retrotransposons, we used non-TIR sequences in the curated library (v6.9.5) to mask TIR-Learner candidates and retain candidates that are longer than 80 bp after removal of masked regions. The test library made of clean candidates is labeled “TIR-Learner_rmLTR” in this study.

To make TIR-Learner universal to non-rice and non-maize species, we collected TIR sequences from various TE databases including Repbase [[7]](https://paperpile.com/c/Ovk0Hl/w23c), P-MITE [[24]](https://paperpile.com/c/Ovk0Hl/dsR1), RiTE databse [[38]](https://paperpile.com/c/Ovk0Hl/7wkW), the Transposable Elements Platform (TREP) [[39]](https://paperpile.com/c/Ovk0Hl/4ZIR), as well as TIRs from rice (our curated library), maize [[40]](https://paperpile.com/c/Ovk0Hl/4paX), and Arabidopsis [[41]](https://paperpile.com/c/Ovk0Hl/RMoe) as training data. Sequences labeled as TIRs that lacked the TIR structure were discarded and then classified to remove redundant elements. After this cleaning step, we randomly selected up to 3,000 TIR elements in each superfamily to train a deep neural network using keras (v2.2.4, <https://github.com/keras-team/keras>). All novel TIR candidates were classified by this general model.

We also tried MITE Digger [[42]](https://paperpile.com/c/Ovk0Hl/uLLn), Repetitive Sequence with Precise Boundaries (RSPB) [[43]](https://paperpile.com/c/Ovk0Hl/6eSw), and iMITEdb [[44]](https://paperpile.com/c/Ovk0Hl/UPgv). MITE Digger is a non-open-source Perl program wrapped as a Windows executable. We encountered Perl/Tk errors when running MITE Digger. RSPB has many steps to obtain MITE candidates, with steps 1-3 being automatic and steps 4-6 requiring extensive manual input. The iMITEdb website (<http://gene.cqu.edu.cn/iMITEdb/>) is not accessible.

*Helitron*

**HelitronScanner** [[45]](https://paperpile.com/c/Ovk0Hl/8L8l) is a Java program that utilizes the local combinational variable (LCV) algorithm to identify sequence patterns that are associated with *Helitron* transposons. The program runs on both positive and negative strands of the input genome, and produces candidates with prediction scores that can help to determine the confidence of the prediction. We followed the guidance provided in the maize B73 v4 annotation [[46]](https://paperpile.com/c/Ovk0Hl/ZKvi) and ran HelitronScanner on entire chromosomes without splitting into chunks (-buffer_size 0). We filtered candidates that are not inserted into AT or TT target sites as suggested by [[47]](https://paperpile.com/c/Ovk0Hl/Bkk2) using the script “format_helitronscanner_out.pl” developed in our EDTA toolkit.

Since *Helitrons* tend to capture sequences during their transposition, non-*Helitron* TE sequences and protein-coding sequences could be present. To remove protein-coding sequences, we used the LINE and TIR element transposase database and the MAKER-P plant protein database [[48]](https://paperpile.com/c/Ovk0Hl/rWDd) included in the LTR_retriever package [[11]](https://paperpile.com/c/Ovk0Hl/CT05) to identify non-*Helitron* coding sequences with blastx. Alignment hits with more than 30 aa were retained as real, and if alignments covered ≥ 70% of a *Helitron* candidate, the entire sequence would be discarded. Removal of protein-coding sequence contamination was done using the script “cleanup_proteins.pl” in our EDTA toolkit. To remove non-*Helitron* TE sequences, we used the non-*Helitron* portion of the curated library to mask *Helitron* candidates. The masked sequences were removed and if the remaining sequence was shorter than 100 bp, then the entire sequence was discarded. If the beginning or ending of candidate sequences were masked, then the entire sequence was also discarded due to uncertainty in recognizing the 5'-TC...CTRR-3' *Helitron* structure. Removal of non-*Helitron* TE contamination was accomplished with the script “cleanup_tandem.pl” in our EDTA toolkit with parameters “-misschar N -nc 50000 -nr 0.9 -minlen 100 -minscore 3000 -trf 1 -cleanN 1 -cleanT 1”. Sequence logos of 10 bp flanking and 30 bp terminal sequences were generated using WebLogo 3 (<http://weblogo.threeplusone.com/>) [[49]](https://paperpile.com/c/Ovk0Hl/yPZn).

We also tried HelitronFinder [[50]](https://paperpile.com/c/Ovk0Hl/XGmY), HelSearch [[51]](https://paperpile.com/c/Ovk0Hl/URMT), and a method previously described by Dong *et al.* (2011) [[52]](https://paperpile.com/c/Ovk0Hl/w73a). HelitronFinder seems to be the predecessor of HelitronScanner, however, we could not find the package. HelSearch is a Perl program that relies on blastall which was extremely slow. We split the rice genome into 12 portions (1 chromosome per file) and ran HelSearch in parallel. After three weeks, none of these jobs were finished and we did not find any *Helitron* candidates. Dong *et al.* (2011) developed Perl scripts for identification of *Helitrons*, however, they are not fully automated and not available for public download.

### Verification of TIR and MITE candidates

TE candidates obtained from detectMITE, GRF-mite_dft, MITE-Hunter, MITE-Tracker, and TIR-Learner were BLAST against the curated library (v6.9.5) to identify any new candidates not contained in the curated library. New candidates were defined as those not confidently matching any sequences in the curated library. Depending on the length of TIRs, short TIRs (i.e., ~10 bp) in these new TIR candidates may still be present in TIR elements in the curated library. We extracted the TIR sequences from new TE candidates based on software output information. For example, in detectMITE, the sequence names are formatted as “>1|6030873|6031020|12|2”, where “12” is the length of the TIR; in GRF, the sequence names are formatted as “>Chr4:7098930:7099636:1m1M10m:TA:3…”, where numbers in “1m1M10m” add up to the length of the TIR (=12). TIR lengths in MITE-Hunter candidates do not have this defined nomenclature to designate the length of the TIR, therefore, 10 bp were extracted from candidate sequences as the minimum length of TIR. TIR sequences were then clustered using the *cd-hit-est-2d* command from CD-HIT [[6]](https://paperpile.com/c/Ovk0Hl/0a2b) with 80% identity threshold. The number of new TIR candidates clustered with the curated library were recorded for each program. For the TIR candidates that possessed novel TIRs, we identified TIR transposon related conserved domains using the NCBI conserved domain database (<https://www.ncbi.nlm.nih.gov/Structure/cdd/>) with default tbastn parameters [[53]](https://paperpile.com/c/Ovk0Hl/rFMe). Redundancy and nesting in novel TIR candidates was removed using the script “cleanup_nested.pl” in our EDTA toolkit, and these non-redundant sequences were used to annotate the rice genome. Copy number of novel TIR candidates in the rice genome were counted by utilizing the script “count_repeats.pl” in our EDTA toolkit. For genomic sequences that cover more than 80% of an TIR candidate, the region was counted as a complete copy, but was otherwise regarded as fragmented.

### Benchmarking comprehensive annotation pipeline

**REPET** (v2.5) [[54]](https://paperpile.com/c/Ovk0Hl/vROr) is a widely used structural based and homology based TE identification and annotation pipeline. REPET is very difficult to install. We have REPET installed on a Compute Canada server on the Arbutus cloud (CentOS6, 120 Gb of RAM and 32 cores) by a team of software engineers. Shell scripts were written to automate the creation of working directories and the running of processes in the pipeline. REPET consists of two separate processes, TEdenovo for repeat library generation and TEannot for genome annotation. To be consistent with other software, only the TEdenovo process was used in this study, while genome annotation was performed using RepeatMasker (v4.0.9). TEdenovo divides the genome into chunks, performs self-alignment of the genome using BLAST (v2.8.1), structural detection of LTR retrotransposons using LTRharvest [[14]](https://paperpile.com/c/Ovk0Hl/Ch9e), clustering of High Scoring Pairs (HSPs) using RECON (v1.08) [[1]](https://paperpile.com/c/Ovk0Hl/xhYU) and PILER (v1.0) [[9]](https://paperpile.com/c/Ovk0Hl/uMnX), repeat consensus building, filtering, and consensus clustering. TEdenovo was run on the rice genome using default parameters with the following exceptions (structural_search: maxLTRSize = 7000, maxElementSize = 25000, LTR_similarity = 85; self_align: identity: 85, min_identity: 85, max_length: 25000; cluster_HSPs: maxSeqLength: 25000; filter_consensus: filter_host_gene = yes). Final consensus clustering was performed by using BLASTClust (v2.2.9) (ftp://[ftp.ncbi.nih.gov/blast/documents/blastclust.html](http://ftp.ncbi.nih.gov/blast/documents/blastclust.html)). REPET could not structurally identify *Helitrons* and there was no *Helitrons* represented in the resulting TE library.

The coding sequences (CDS) obtained from the MSU Rice Genome Annotation Project (MSUv7) were used to filter potential gene sequences in the TE library. The loci with FPKM levels 10 or greater based on the v7 expression matrix file were used for this cleaning process. Loci identified as derived from transposons and retrotransposons (indicated in the sequence name) were also removed.

**Tephra** (<https://github.com/sestaton/tephra>) is a command line application that combines multiple structure-based programs for comprehensive TE annotation [[55]](https://paperpile.com/c/Ovk0Hl/j0Ze). These programs include LTRharvest, LTRdigest, HelitronScanner, TIRvish, and MGEScan_nonLTR. Tephra employs a sequential identification-and-mask strategy for different TE types, in which it first identifies LTR retrotransposons, masks them in the genomic sequence, and then iteratively identifies *Helitrons*, TIR elements, and non-LTR retrotransposons with the same process in this order. This application is available as both a *docker* image and by manual installation versions (accessed 07/16/2019), both of which require *sudo* privileges for installation and usage. At the time we were summarizing the results (08/16/2019), the singularity version was made available per our request, which does not require *sudo* privileges and is necessary for some HPC users.

To benchmark tephra, we installed the *docker* version in an iMac machine and ran the program on each rice chromosome separately, as the high memory load exceeded the memory capacity of the machine when running across the entire genome (8 Gb). The resulting libraries from each chromosome were aggregated and tandem repeats and gapped sequences were removed using the script “cleanup_tandem.pl” in our EDTA toolkit. Redundancy in the aggregated library was reduced with five rounds of iterations using the script “cleanup_nested.pl” in our EDTA toolkit. The resulting file was used as the test library for benchmarking of tephra.

**EDTA** is developed to be an accurate, sensitive, and handy tool for TE annotations of eukaryotic genomes. To demonstrate these features, we benchmarked EDTA in the rice (MSU7), maize (AGPv4), and Drosophila (r6.28) genome for both annotation and computational performance. Our manually curated rice TE library (v6.9.5) served as the standard for benchmarking in rice. The Maize TE Consortium (MTEC) library (<http://maizetedb.org/~maize>) and the Repbase-Drosophila library (version 20170127) were both manually curated by respective communities and served as the standard library for benchmarking in maize and Drosophila, respectively. The three genomes were run with default EDTA parameters except for the *-species* parameter specifying “rice”, “maize”, and “others” respectively for the identification of TIR elements. The resulting TE libraries from the EDTA pipeline are cleaned and have had redundancy reduced, and were used as is for the subsequent benchmarking.

*Misclassification*

**Misclassification** is an important issue for TE annotations. A misclassified library sequence could lead to massive misannotation of TE sequences in the genome. There are two types of misclassifications: full-length misclassifications and misclassification of sequence that results from nested insertions. Here, we defined full-length misclassification as having different copies of the same TE sequence inconsistently classified as different TE types. In this case, at least one of the inconsistently classified elements is misclassified. Nested insertions is the case when one or more TEs are inserted into another TE. If the nested TE(s) are not identified, the entire sequence would be classified as one element, and therefore one element type. When the nested TE type is different from that of the target TE type, the sequence from the nested element is misclassified.

To quantify misclassifications during whole-genome TE annotations, we extracted all TE sequences in the genome that were annotated using the focal TE library. Each sequence (query) was blasted against each of the other TE sequences (subject). If ≥ 95% of the query sequence could be aligned with a subject, it was counted as a full query match. For a full query match, if ≥ 95% of the subject was also covered by the query, then the subject was classified as a redundant copy, otherwise the subject was classified as a nested TE. Redundant subjects were removed in the sequence pool, while the query-matching regions in nested TE subjects were removed and the remaining sequences were joined. If the joined new sequence was less than 80 bp, the sequence would be discarded, otherwise it was used to replace the original subject in the sequence pool. This complete process was iterated automatically to thoroughly remove multi-layer nested insertions. Since each TE sequence in the whole genome annotation inherits the classification from the annotating library sequence, misclassifications for each TE type in the whole-genome annotation could be determined without the need of a curated standard. Identification and quantification of TE misclassifications were achieved using the scripts “cleanup_nested.pl” and “count_nested.pl” in our EDTA toolkit, respectively.

## References

[1. Bao Z, Eddy SR. Automated *de novo* identification of repeat sequence families in sequenced genomes. Genome Res. 2002;12:1269–76.](http://paperpile.com/b/Ovk0Hl/xhYU)

[2. Price AL, Jones NC, Pevzner PA. *De novo* identification of repeat families in large genomes. Bioinformatics. 2005;21 Suppl 1:i351–8.](http://paperpile.com/b/Ovk0Hl/PObp)

[3. Smit AFA, Hubley R. RepeatModeler Open-1.0. 2008--2015 [Internet]. 2015. Available from:](http://paperpile.com/b/Ovk0Hl/8ZJO) [www.repeatmasker.org](http://www.repeatmasker.org)

[4. Girgis HZ. Red: an intelligent, rapid, accurate tool for detecting repeats *de-novo* on the genomic scale. BMC Bioinformatics. 2015;16:227.](http://paperpile.com/b/Ovk0Hl/1SNe)

[5. Shi J, Liang C. Generic Repeat Finder: a high-sensitivity tool for genome-wide de novo repeat detection. Plant Physiology. 2019;180:1803–15.](http://paperpile.com/b/Ovk0Hl/ByTu)

[6. Fu L, Niu B, Zhu Z, Wu S, Li W. CD-HIT: accelerated for clustering the next-generation sequencing data. Bioinformatics. 2012;28:3150–2.](http://paperpile.com/b/Ovk0Hl/0a2b)

[7. Bao W, Kojima KK, Kohany O. Repbase Update, a database of repetitive elements in eukaryotic genomes. Mob DNA. 2015;6:11.](http://paperpile.com/b/Ovk0Hl/w23c)

[8. Kohany O, Gentles AJ, Hankus L, Jurka J. Annotation, submission and screening of repetitive elements in Repbase: RepbaseSubmitter and Censor. BMC Bioinformatics. 2006;7:474.](http://paperpile.com/b/Ovk0Hl/PJZJ)

[9. Edgar RC, Myers EW. PILER: identification and classification of genomic repeats. Bioinformatics. 2005;21 Suppl 1:i152–8.](http://paperpile.com/b/Ovk0Hl/uMnX)

[10. McCarthy EM, McDonald JF. LTR_STRUC: a novel search and identification program for LTR retrotransposons. Bioinformatics. 2003;19:362–7.](http://paperpile.com/b/Ovk0Hl/SxBQ)

[11. Ou S, Jiang N. LTR_retriever: A Highly Accurate and Sensitive Program for Identification of Long Terminal Repeat Retrotransposons. Plant Physiol. 2018;176:1410–22.](http://paperpile.com/b/Ovk0Hl/CT05)

[12. Xu Z, Wang H. LTR_FINDER: an efficient tool for the prediction of full-length LTR retrotransposons. Nucleic Acids Res. 2007;35:W265–8.](http://paperpile.com/b/Ovk0Hl/nwsc)

[13. Ou S, Jiang N. LTR_FINDER_parallel: parallelization of LTR_FINDER enabling rapid identification of long terminal repeat retrotransposons [Internet]. bioRxiv. 2019 [cited 2019 Aug 17]. p. 722736. Available from:](http://paperpile.com/b/Ovk0Hl/bpra) <https://www.biorxiv.org/content/10.1101/722736v1>

[14. Ellinghaus D, Kurtz S, Willhoeft U. LTRharvest, an efficient and flexible software for *de novo* detection of LTR retrotransposons. BMC Bioinformatics. 2008;9:18.](http://paperpile.com/b/Ovk0Hl/Ch9e)

[15. Lee H, Lee M, Mohammed Ismail W, Rho M, Fox GC, Oh S, et al. MGEScan: a Galaxy-based system for identifying retrotransposons in genomes. Bioinformatics. 2016;32:2502–4.](http://paperpile.com/b/Ovk0Hl/sED5)

[16. Rho M, Schaack S, Gao X, Kim S, Lynch M, Tang H. LTR retroelements in the genome of *Daphnia pulex*. BMC Genomics. 2010;11:425.](http://paperpile.com/b/Ovk0Hl/I4QL)

[17. Rho M, Tang H. MGEScan-non-LTR: computational identification and classification of autonomous non-LTR retrotransposons in eukaryotic genomes. Nucleic Acids Res. 2009;37:e143.](http://paperpile.com/b/Ovk0Hl/ZPaW)

[18. Valencia JD, Girgis HZ. LtrDetector: A tool-suite for detecting long terminal repeat retrotransposons de-novo. BMC Genomics. 2019;20:450.](http://paperpile.com/b/Ovk0Hl/KQgL)

[19. Pereira V. Insertion bias and purifying selection of retrotransposons in the *Arabidopsis thaliana* genome. Genome Biol. 2004;5:R79.](http://paperpile.com/b/Ovk0Hl/cN8z)

[20. You FM, Cloutier S, Shan Y, Ragupathy R. LTR annotator: automated identification and annotation of LTR retrotransposons in plant genomes. Int J Biosci Biochem Bioinforma. IACSIT Press; 2015;5:165.](http://paperpile.com/b/Ovk0Hl/ECgZ)

[21. Vassetzky NS, Kramerov DA. SINEBase: a database and tool for SINE analysis. Nucleic Acids Res. 2013;41:D83–9.](http://paperpile.com/b/Ovk0Hl/tiOB)

[22. Wenke T, Döbel T, Sörensen TR, Junghans H, Weisshaar B, Schmidt T. Targeted identification of short interspersed nuclear element families shows their widespread existence and extreme heterogeneity in plant genomes. Plant Cell. 2011;23:3117–28.](http://paperpile.com/b/Ovk0Hl/t2OV)

[23. Mao H, Wang H. SINE_scan: an efficient tool to discover short interspersed nuclear elements (SINEs) in large-scale genomic datasets. Bioinformatics. 2017;33:743–5.](http://paperpile.com/b/Ovk0Hl/qQFq)

[24. Chen J, Hu Q, Zhang Y, Lu C, Kuang H. P-MITE: a database for plant miniature inverted-repeat transposable elements. Nucleic Acids Res. 2014;42:D1176–81.](http://paperpile.com/b/Ovk0Hl/dsR1)

[25. Warburton PE, Giordano J, Cheung F, Gelfand Y, Benson G. Inverted repeat structure of the human genome: the X-chromosome contains a preponderance of large, highly homologous inverted repeats that contain testes genes. Genome Res. 2004;14:1861–9.](http://paperpile.com/b/Ovk0Hl/5STs)

[26. Han Y, Wessler SR. MITE-Hunter: a program for discovering miniature inverted-repeat transposable elements from genomic sequences. Nucleic Acids Res. 2010;38:e199.](http://paperpile.com/b/Ovk0Hl/Aa1t)

[27. Gremme G, Steinbiss S, Kurtz S. GenomeTools: a comprehensive software library for efficient processing of structured genome annotations. IEEE/ACM Trans Comput Biol Bioinform. 2013;10:645–56.](http://paperpile.com/b/Ovk0Hl/ZD7K)

[28. Ye C, Ji G, Liang C. detectMITE: A novel approach to detect miniature inverted repeat transposable elements in genomes. Sci Rep. 2016;6:19688.](http://paperpile.com/b/Ovk0Hl/UxsV)

[29. Zhao D, Ferguson AA, Jiang N. What makes up plant genomes: The vanishing line between transposable elements and genes. Biochim Biophys Acta. 2016;1859:366–80.](http://paperpile.com/b/Ovk0Hl/tWbe)

[30. Hu J, Zheng Y, Shang X. MiteFinderII: a novel tool to identify miniature inverted-repeat transposable elements hidden in eukaryotic genomes. BMC Med Genomics. 2018;11:101.](http://paperpile.com/b/Ovk0Hl/1Kjh)

[31. Crescente JM, Zavallo D, Helguera M, Vanzetti LS. MITE Tracker: an accurate approach to identify miniature inverted-repeat transposable elements in large genomes. BMC Bioinformatics. 2018;19:348.](http://paperpile.com/b/Ovk0Hl/rf9h)

[32. Rognes T, Flouri T, Nichols B, Quince C, Mahé F. VSEARCH: a versatile open source tool for metagenomics. PeerJ. 2016;4:e2584.](http://paperpile.com/b/Ovk0Hl/eNIp)

[33. Ge R, Mai G, Zhang R, Wu X, Wu Q, Zhou F. MUSTv2: An Improved *De Novo* Detection Program for Recently Active Miniature Inverted Repeat Transposable Elements (MITEs). J Integr Bioinform. 2017;14:20170029.](http://paperpile.com/b/Ovk0Hl/OUyw)

[34. Kent WJ. BLAT—The BLAST-Like Alignment Tool. Genome Res. 2002;12:656–64.](http://paperpile.com/b/Ovk0Hl/Wkpl)

[35. Stajich JE, Block D, Boulez K, Brenner SE, Chervitz SA, Dagdigian C, et al. The Bioperl toolkit: Perl modules for the life sciences. Genome Res. 2002;12:1611–8.](http://paperpile.com/b/Ovk0Hl/M20w)

[36. Su W, Gu X, Peterson T. TIR-Learner, a New Ensemble Method for TIR Transposable Element Annotation, Provides Evidence for Abundant New Transposable Elements in the Maize Genome. Mol Plant. 2019;12:447–60.](http://paperpile.com/b/Ovk0Hl/WdzE)

[37. Feschotte C, Pritham EJ. DNA transposons and the evolution of eukaryotic genomes. Annu Rev Genet. 2007;41:331–68.](http://paperpile.com/b/Ovk0Hl/hMnn)

[38. Copetti D, Zhang J, El Baidouri M, Gao D, Wang J, Barghini E, et al. RiTE database: a resource database for genus-wide rice genomics and evolutionary biology. BMC Genomics. 2015;16:538.](http://paperpile.com/b/Ovk0Hl/7wkW)

[39. Wicker T, Sabot F, Hua-Van A, Bennetzen JL, Capy P, Chalhoub B, et al. A unified classification system for eukaryotic transposable elements. Nature Reviews Genetics. 2007;8:973–82.](http://paperpile.com/b/Ovk0Hl/4ZIR)

[40. Schnable PS, Ware D, Fulton RS, Stein JC, Wei F, Pasternak S, et al. The B73 maize genome: complexity, diversity, and dynamics. Science. 2009;326:1112–5.](http://paperpile.com/b/Ovk0Hl/4paX)

[41. The Arabidopsis Genome Initiative. Analysis of the genome sequence of the flowering plant Arabidopsis thaliana. Nature. 2000;408:796–815.](http://paperpile.com/b/Ovk0Hl/RMoe)

[42. Yang G. MITE Digger, an efficient and accurate algorithm for genome wide discovery of miniature inverted repeat transposable elements. BMC Bioinformatics. 2013;14:186.](http://paperpile.com/b/Ovk0Hl/uLLn)

[43. Lu C, Chen J, Zhang Y, Hu Q, Su W, Kuang H. Miniature Inverted-Repeat Transposable Elements (MITEs) Have Been Accumulated through Amplification Bursts and Play Important Roles in Gene Expression and Species Diversity in *Oryza sativa*. Molecular Biology and Evolution. 2012;29:1005–17.](http://paperpile.com/b/Ovk0Hl/6eSw)

[44. Han M-J, Zhou Q-Z, Zhang H-H, Tong X, Lu C, Zhang Z, et al. iMITEdb: the genome-wide landscape of miniature inverted-repeat transposable elements in insects. Database. 2016;2016:baw148.](http://paperpile.com/b/Ovk0Hl/UPgv)

[45. Xiong W, He L, Lai J, Dooner HK, Du C. HelitronScanner uncovers a large overlooked cache of *Helitron* transposons in many plant genomes. Proc Natl Acad Sci U S A. 2014;111:10263–8.](http://paperpile.com/b/Ovk0Hl/8L8l)

[46. Jiao Y, Zhao H, Ren L, Song W, Zeng B, Guo J, et al. Genome-wide genetic changes during modern breeding of maize. Nat Genet. 2012;44:812–5.](http://paperpile.com/b/Ovk0Hl/ZKvi)

[47. Thomas J, Pritham EJ. Helitrons, the Eukaryotic Rolling-circle Transposable Elements. Microbiol Spectr. 2015;3:1–32.](http://paperpile.com/b/Ovk0Hl/Bkk2)

[48. Campbell MS, Holt C, Moore B, Yandell M. Genome annotation and curation using MAKER and MAKER-P. Curr Protoc Bioinformatics. Wiley Online Library; 2014;48:4–11.](http://paperpile.com/b/Ovk0Hl/rWDd)

[49. Crooks GE, Hon G, Chandonia J-M, Brenner SE. WebLogo: a sequence logo generator. Genome Res. 2004;14:1188–90.](http://paperpile.com/b/Ovk0Hl/yPZn)

[50. Du C, Caronna J, He L, Dooner HK. Computational prediction and molecular confirmation of *Helitron* transposons in the maize genome. BMC Genomics. 2008;9:51.](http://paperpile.com/b/Ovk0Hl/XGmY)

[51. Yang L, Bennetzen JL. Structure-based discovery and description of plant and animal Helitrons. Proc Natl Acad Sci U S A. 2009;106:12832–7.](http://paperpile.com/b/Ovk0Hl/URMT)

[52. Dong Y, Lu X, Song W, Shi L, Zhang M, Zhao H, et al. Structural characterization of *helitrons* and their stepwise capturing of gene fragments in the maize genome. BMC Genomics. 2011;12:609.](http://paperpile.com/b/Ovk0Hl/w73a)

[53. Marchler-Bauer A, Derbyshire MK, Gonzales NR, Lu S, Chitsaz F, Geer LY, et al. CDD: NCBI’s conserved domain database. Nucleic Acids Res. 2015;43:D222–6.](http://paperpile.com/b/Ovk0Hl/rFMe)

[54. Flutre T, Duprat E, Feuillet C, Quesneville H. Considering transposable element diversification in *de novo* annotation approaches. PLoS One. 2011;6:e16526.](http://paperpile.com/b/Ovk0Hl/vROr)

[55. Badouin H, Gouzy J, Grassa CJ, Murat F, Staton SE, Cottret L, et al. The sunflower genome provides insights into oil metabolism, flowering and Asterid evolution. Nature. 2017;546:148–52.](http://paperpile.com/b/Ovk0Hl/j0Ze)
