# Supplementary material for: Benchmarking transposable element annotation methods for creation of a streamlined, comprehensive pipeline
Source: Genome Biol. 2019 Dec 16;20:275. doi: 10.1186/s13059-019-1905-y (PMC6913007; doi:10.1186/s13059-019-1905-y)
Supplement: Supplementary file 3 — Additional file 3: Table S3. Verification of new TIR candidates identified by TIR-Learner and MITE programs. Table S4. Comparison of whole-genome Helitron annotations using the curated library (v6.9.5), the HelitronScanner clean library (HS_clean), and the EDTA filtered HelitronScanner library (HS_EDTA). Table S5. TE content in the maize (Zea mays cv. ‘B73’ v. 4) genome. Table S6. TE content in the Drosophila (Drosophila melanogaster r6.28) genome. Figure S1. Sequence logos of terminal and flanking sequences of Helitron candidates cleaned by the standard library. Figure S2. Performance of TE annotation programs. [file 13059_2019_1905_MOESM3_ESM.docx]

## Additional File 3. Supplementary Figures and Tables

**Table S3:** Verification of new TIR candidates identified by TIR-Learner and MITE programs.

| Software | New TE | New TE with known TIRs | Unique new TE with new TIRs | New TIRs with conserved domains* | New TIRs with copy number > 3 |
| --- | --- | --- | --- | --- | --- |
| detectMITE | 15,654 | 10,947 | 1,341 | 20 | 1,018 |
| GRF-mite_dft | 1,489 | 687 | 354 | 38 | 331 |
| MITE-Hunter | 114 | 114 | 0 | - | - |
| MITE-Tracker | 836 | 137 | 668 | 34 | 577 |
| TIR-Learner | 7,435 | 5,688 | 1,629 | 51 | 331 |

*Conserved protein domains from known TIR elements were used to annotate new TIR candidates.

**Table S4:** Comparison of whole-genome *Helitron* annotations using the curated library (v6.9.5), the HelitronScanner clean library (HS_clean), and the EDTA filtered HelitronScanner library (HS_EDTA).

|  | Std 6.9.5 | HS_clean | HS_EDTA |
| --- | --- | --- | --- |
| Fragment # | 39,640 | 481,927 | 45,266 |
| Mean length | 340 bp | 183 bp | 606 bp |
| Median length | 159 bp | 116 bp | 301 bp |
| Total size | 13.3 Mb | 64.3 Mb | 27.4 Mb |
| Genome % | 3.57% | 17.16% | 7.33% |

**Table S5:** TE content in the maize (*Zea mays* cv. ‘B73’ v. 4) genome.

|  | Class | MTEC* | Complete** | Fragmented** | Total** |
| --- | --- | --- | --- | --- | --- |
| LTR | Class I | 1571.0 Mb | 27.74% | 45.87% | 73.61% |
| non-LTR | Class I | 15.3 Mb | 0.10% | 0.62% | 0.72% |
| TIR | Class II | 131.6 Mb | 1.74% | 4.43% | 6.16% |
| *Helitron* | Class II | 7.8 Mb | 0.08% | 0.29% | 0.37% |
| Total | - | 1725.7 Mb | 29.65% | 51.20% | 80.85% |

* Annotation based on the curated library (MTEC maizeTE10102014).

** Percent of genome estimated based on a genome size of 2134.4 Mb.

**Table S6:** TE content in the Drosophila (*Drosophila melanogaster* r6.28) genome.

|  | Class | RepBase* | Complete** | Fragmented** | Total** |
| --- | --- | --- | --- | --- | --- |
| LTR | Class I | 14.48 Mb | 3.85% | 6.31% | 10.16% |
| non-LTR | Class I | 5.59 Mb | 1.03% | 2.89% | 3.92% |
| TIR | Class II | 1.88 Mb | 0.46% | 0.86% | 1.32% |
| *Helitron* | Class II | 0.97 Mb | 0.12% | 0.56% | 0.68% |
| Unknown | Unknown | 1.46 Mb | 0.13% | 0.89% | 1.03% |
| Total | - | 24.4 Mb | 5.59% | 11.51% | 17.11% |

* Annotation based on the curated library (RepBase 20170127).

** Percent of genome estimated based on a genome size of 142.6 Mb.


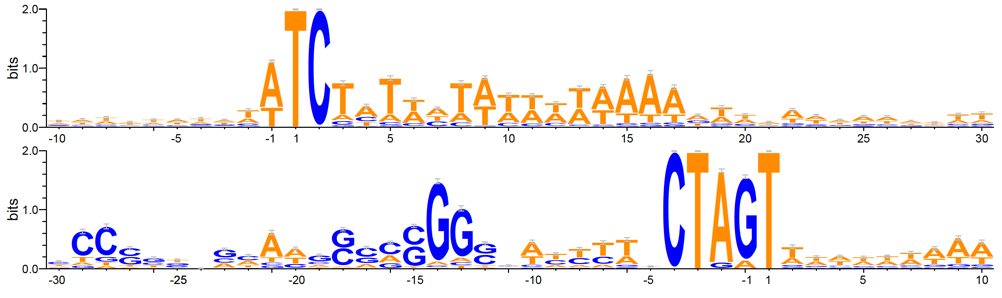


**Fig. S1.** Sequence logos of terminal and flanking sequences of *Helitron* candidates cleaned by the standard library. Upper panel, 5' terminal of *Helitron*-candidates with the starting position labeled as 1; Lower panel, 3' terminal of *Helitron*-candidates with the last position labeled as -1.


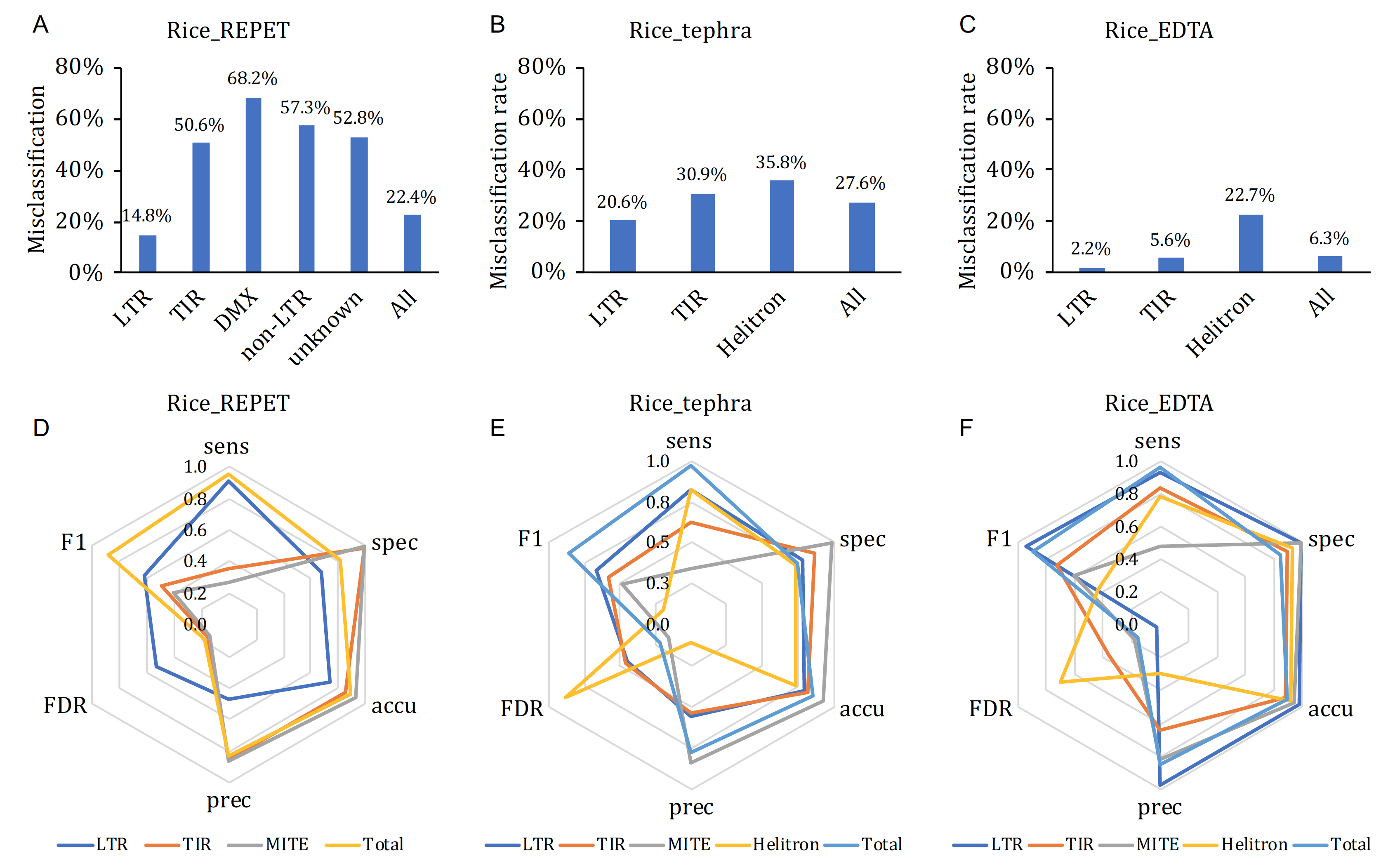


**Fig. S2.** Performance of TE annotation programs. (A-C) Misclassifications were calculated based on TE annotations using the program-produced TE library on the rice genome. (D-F) The TE annotation generated with the rice curated TE library (v6.9.5) was used as the standard for benchmarking.
