# Supplementary material for: Benchmarking transposable element annotation methods for creation of a streamlined, comprehensive pipeline
Source: Genome Biol. 2019 Dec 16;20:275. doi: 10.1186/s13059-019-1905-y (PMC6913007; doi:10.1186/s13059-019-1905-y)
Supplement: Supplementary file 4 — Additional file 4. Review history. [file 13059_2019_1905_MOESM4_ESM.docx]

Review History

**First round of review**

**Reviewer 1**

**Are you able to assess all statistics in the manuscript, including the appropriateness of statistical tests used?**

No, I do not feel adequately qualified to assess the statistics.

**Comments to author:**

The authors benchmarked existing programs based on a curated library of rice TEs, and further created a  pipeline called Extensive de-novo TE Annotator (EDTA) that produces a condensed TE library for annotations of structurally intact and fragmented elements. The methods and testing used by the authors look comprehensive and give a direction for best possible ways of TE annotation. The pipeline should have a potential to be used broadly. However, since the purpose of the work is to provide a pipeline for TE annotation for general usage, I would like to see the results of TE annotation on another genome such as maize from EDTA and comparison to previously published annotations.

minor issues:
1. Optimal is a too strong word, should use a more suitable one.
The manuscript can be shortened to make it more concise, eg, first two paragraph in Results.

**Reviewer 2**

**Are you able to assess all statistics in the manuscript, including the appropriateness of statistical tests used?**

No, I do not feel adequately qualified to assess the statistics.

**Comments to author:**

 Authors compared current programs of TEs annotation, selected the robust programs and created a pipeline EDTA. They used integrated environment Conda for installation of tools which is helpful and easy to use. In this study they benchmark their process with a relatively small, well studied and less repetitive Rice genome. It would be more intuitive for readers to understand the performance of their method if the specific numbers of sensitivity, efficiency and accuracy of EDTA could be presented with relatively larger genomes including Maize, Wheat or Cotton. The results need to be compared with those reported in the manuscript, just as what they have done for rice. It would be interesting if the differences between them could be fully discussed. If EDTA can handle bigger and highly repetitive genomes, it will be much more powerful. I understand this means significant amount of extra work but without this current findings of this manuscript have not enough potential to be considered for publication in Genome Biology.
     In addition, all the figures are rough, in Figure 2A, the sensitivity, specificity, 1.000,  0.800, etc  are used, but in other figures, they use 1.0, 0.8, etc. The table in Fig. 5D should be separated tables and not in the Figure.

**Authors Response**

**Point-by-point responses to the reviewers’ comments:**

Reviewer #1:

The authors benchmarked existing programs based on a curated library of rice TEs, and further created a pipeline called Extensive de-novo TE Annotator (EDTA) that produces a condensed TE library for annotations of structurally intact and fragmented elements. The methods and testing used by the authors look comprehensive and give a direction for best possible ways of TE annotation. The pipeline should have a potential to be used broadly. However, since the purpose of the work is to provide a pipeline for TE annotation for general usage, I would like to see the results of TE annotation on another genome such as maize from EDTA and comparison to previously published annotations.

*Response: Thank you for affirming our benchmarking methods and the potential impact of EDTA. We agree that EDTA should be demonstrated as robust across other genomes such as maize. We applied the EDTA pipeline to the maize genome and compared the EDTA annotation to the community-curated TE library, the Maize TE Consortium (MTEC) library. In maize, we see low misclassification rates, high sensitivity, high specificity, and high accuracy, results comparable to those obtained in rice (Figure 6). We also improved multiple time-intensive steps in the EDTA pipeline, so that it could be executed within a reasonable timeframe for large genomes such as maize. After these improvements, it took less than a week to annotate the maize genome using EDTA. To demonstrate its application on non-plant species, we benchmarked the EDTA pipeline with the Drosophila genome which also has a high-quality TE annotation. Again, EDTA demonstrated consistently high performance comparable to rice and maize results (Figure 6).*

minor issues:
1. Optimal is a too strong word, should use a more suitable one.
The manuscript can be shortened to make it more concise, eg, first two paragraph in Results.

*Response: Thank you for the suggestions. We have reworded “optimal” to “comprehensive” in the revised manuscript. We have also shortened the first two paragraphs in the Results.*

Reviewer #2:

Authors compared current programs of TEs annotation, selected the robust programs and created a pipeline EDTA. They used integrated environment Conda for installation of tools which is helpful and easy to use. In this study they benchmark their process with a relatively small, well studied and less repetitive Rice genome. It would be more intuitive for readers to understand the performance of their method if the specific numbers of sensitivity, efficiency and accuracy of EDTA could be presented with relatively larger genomes including Maize, Wheat or Cotton. The results need to be compared with those reported in the manuscript, just as what they have done for rice. It would be interesting if the differences between them could be fully discussed. If EDTA can handle bigger and highly repetitive genomes, it will be much more powerful. I understand this means significant amount of extra work but without this current findings of this manuscript have not enough potential to be considered for publication in Genome Biology.

*Response: Thank you for confirming the ease of use of EDTA. We have strived to provide an easy yet high-quality method to facilitate TE annotation. We agree that EDTA should be benchmarked in larger and highly repetitive genomes. Benchmarking requires a manually curated standard which is often unavailable. Fortunately, maize has this resource and we thus tested the performance of EDTA in this genome. Please refer to our response to Reviewer #1 for more details. We carefully read the genome papers of wheat and cotton and consulted with experts studying the two species and found both species lack careful TE curation, making them unacceptable as standard species for benchmarking our pipeline. Thus, we were not able to benchmark the EDTA pipeline in these two genomes. However, we did also test the EDTA pipeline using a non-plant species (Drosophila) that did have a well-curated TE library and observed performance metrics that were consistent with those in plant species (rice and maize).*

In addition, all the figures are rough, in Figure 2A, the sensitivity, specificity, 1.000, 0.800, etc are used, but in other figures, they use 1.0, 0.8, etc. The table in Fig. 5D should be separated tables and not in the Figure.

*Response: Thank you for this suggestion. We have improved the quality of our figures and have improved consistency. We have corrected the coordinates in Figure 2A. We also moved tables in Figure 5D and Figure 6B to Supplementary Table S3 and Supplementary Table S4, respectively. Also, we combined Figure 3 and Figure 4 into what is now Figure 3, and Figure 5 and Figure 6 into what is now Figure 4 to streamline the manuscript.*

**Second round of review**

**Reviewer 1**

The authors took efforts to add evaluation on maize and Drosophila genomes. However, for users to have better understanding on the performance of the program/pipeline, they should provide more details on the repeat data they have obtained in maize and Drosophila as summarized for rice in Table 1. The sensitivity looks not very high. Therefore, they should also clearly stated the standard data they used for evaluation, and the overall results for the whole genome, so that users can get an idea whether their annotation is complete. The completeness of repeat annotation is critical for a new geome. Ideally they can also provide such a summary for wheat, so that users can understand more by comparing the number to existing annotations. For the annotations differing from the standard library or existing annotations, the authors can randomly select a number of (say 20) elements to examine which annotation is correct.

**Reviewer 2**

All required questions have been answered and that all responses meet formatting specifications.

**Authors Response**

**Point-by-point responses to the reviewers’ comments:**

***The authors took efforts to add evaluation on maize and Drosophila genomes. However, for users to have better understanding on the performance of the program/pipeline, they should provide more details on the repeat data they have obtained in maize and Drosophila as summarized for rice in Table 1. The sensitivity looks not very high. Therefore, they should also clearly stated the standard data they used for evaluation, and the overall results for the whole genome, so that users can get an idea whether their annotation is complete.***

*We have provided summaries of misclassification rates and additional benchmarking metrics for maize and Drosophila in Figure 6 of our manuscript. We also added summary tables (Table S5 and S6) for standard annotations of both the maize and Drosophila genome. While EDTA performs, for the most part, similarly in these additional species to what was observed in rice, we do see a drop in sensitivity in the annotation of Helitrons in both species and in total annotated TEs in Drosophila. We applied stringent filters on Helitrons given their less clear and consistent structural features relative to other classes of TEs and had only one Helitron for benchmarking purposes in the manually curated library for Drosophila. We additionally saw low specificity in the total TE annotations in Drosophila because TE fragments were manually identified and annotated in the exemplar library (Table S6). Such fragments have no corresponding intact elements in the genome. Thus, EDTA cannot identify these fragments based on the library constructed from intact elements.*

***The completeness of repeat annotation is critical for a new genome. Ideally they can also provide such a summary for wheat, so that users can understand more by comparing the number to existing annotations. ***

*We agree with the reviewer completely regarding the importance of complete (or near complete in balancing overly high false positive rates) repeat annotation of new genomes for future biological inferences to be made. In this manuscript we benchmarked existing annotation software for each class of TEs based on their ability to reproduce the results of highly manually curated exemplar libraries. Without the existence of a manually curated library this benchmarking is not possible as there is no standard to which to compare. We extended the benchmarking exercise to develop a comprehensive annotation pipeline that does structural annotation using the best software from the benchmarking, followed by filtering and cleaning steps to remove non TE sequences and produce a high quality TE library without extensive manual annotation efforts. We originally benchmarked the different software programs against three different species with variable TE composition and also tested our final EDTA pipeline against two existing pipelines that were developed for this same purpose. We feel that annotating subsequent new genomes such as wheat, which lacks an exemplar library, is outside of the scope of this study in which the goal was to perform benchmarking and then develop an analysis pipeline using the best available programs.*

***For the annotations differing from the standard library or existing annotations, the authors can randomly select a number of (say 20) elements to examine which annotation is correct.***

*One of the encouraging results emerging from our EDTA pipeline is that there is substantial overlap between the set of TEs annotated in the exemplar libraries and in EDTA. This can be seen in the following table:

Species Class of Element Total intact Represented in the curated library New
Rice DTA 2144 2062 82
Rice DTC 1230 1014 216
Rice DTH 1640 1594 46
Rice DTM 7709 7010 699
Rice DTT 6125 5825 300
Rice DHH (Helitron) 2949 535 2414
Rice LTR 2005 1998 7
Maize LTR 38727 38704 23

Unfortunately, this leaves us, in many cases, with few novel TEs to curate and, based on our inspection of a subset of these, the non-overlapping set of TEs between EDTA and the manually curated libraries are often not high confidence annotations or are single copy within the genome. However, for superfamilies such as DTA, DTH, DTT, DHH, we do identify a number of clear, novel TEs using the EDTA pipeline that have been missed through exhaustive manual curation. These results suggest our automated pipeline based on structural features is quite robust. We have attached a supplementary table listing the curation results in detail (Supplementary Note).*

**Third round of review**

**Reviewer 1**

The authors have addressed my major concerns. I have no more questions.
